# Environmental patterns of brown moss- and *Sphagnum*-associated microbial communities

**DOI:** 10.1038/s41598-020-79773-2

**Published:** 2020-12-29

**Authors:** Alexander Tøsdal Tveit, Andrea Kiss, Matthias Winkel, Fabian Horn, Tomáš Hájek, Mette Marianne Svenning, Dirk Wagner, Susanne Liebner

**Affiliations:** 1 UiT The Arctic University of Norway, Department of Arctic and Marine Biology, Tromsø, Norway; 2 GFZ German Research Center for Geosciences, Section Geomicrobiology, Potsdam, Germany; 3 University of South Bohemia, Faculty of Science, České Budějovice, Czech Republic; 4 University of Potsdam, Institute of Geosciences, Potsdam, Germany; 5 University of Potsdam, Institute of Biochemistry and Biology, Potsdam, Germany

**Keywords:** Environmental microbiology, Microbiology, Environmental sciences

## Abstract

Northern peatlands typically develop through succession from fens dominated by the moss family Amblystegiaceae to bogs dominated by the moss genus *Sphagnum*. How the different plants and abiotic environmental conditions provided in Amblystegiaceae and *Sphagnum* peat shape the respective moss associated microbial communities is unknown. Through a large-scale molecular and biogeochemical study spanning Arctic, sub-Arctic and temperate regions we assessed how the endo- and epiphytic microbial communities of natural northern peatland mosses relate to peatland type (*Sphagnum* and Amblystegiaceae), location, moss taxa and abiotic environmental variables. Microbial diversity and community structure were distinctly different between Amblystegiaceae and *Sphagnum* peatlands, and within each of these two peatland types moss taxon explained the largest part of microbial community variation. *Sphagnum* and Amblystegiaceae shared few (< 1% of all operational taxonomic units (OTUs)) but strikingly abundant (up to 65% of relative abundance) OTUs. This core community overlapped by one third with the *Sphagnum*-specific core-community. Thus, the most abundant microorganisms in *Sphagnum* that are also found in all the *Sphagnum* plants studied, are the same OTUs as those few shared with Amblystegiaceae. Finally, we could confirm that these highly abundant OTUs were endophytes in *Sphagnum*, but epiphytes on Amblystegiaceae. We conclude that moss taxa and abiotic environmental variables associate with particular microbial communities. While moss taxon was the most influential parameter, hydrology, pH and temperature also had significant effects on the microbial communities. A small though highly abundant core community is shared between *Sphagnum* and Amblystegiaceae.

## Introduction

The majority of global wetlands are peatlands (70%)^1^, of which 80% are natural and pristine^2^. Due to the high water content, low temperatures^3^, anoxia and the production of recalcitrant organic matter and microbial inhibitors (e.g., polyphenols)^4^, dead organic matter in northern peatlands decomposes slowly. Almost 20% of the global soil organic carbon and 10% of the global freshwater reservoirs are stored in peatlands^5^. Thus, these ecosystems fulfil crucial functions like maintaining regional water balance and sequestering carbon^6,7^. The vegetation of natural peatlands is dominated by bryophytes of the Amblystegiaceae family (brown mosses) and of the genus *Sphagnum*. Both moss types play an important role in succession and peat formation. Amblystegiaceae initialize peat formation by colonizing open water bodies (‘terrestrialisation’), which leads to the formation of minerotrophic fens. Over time, *Sphagnum* species establish and contribute to peat accumulation (‘paludification’), transforming those fens into ombrotrophic bogs^8–12^. The transition from a fen to an ombrotrophic bog profoundly alters the ecosystem carbon budget due to doubled net primary productivity and a fourfold decrease in the decomposition rate, causing a several fold increase in the peat accumulation rate^13^. Such shifts especially in vegetation have been observed to coincide with drops in pH from 7 to around 4^14–16^.

Microorganisms play a crucial role in the C and N cycles of peatlands^17–19^. Moss-associated microbial communities are suggested to maintain host plant health and pathogen defence^20–24^. In addition, moss shoots, specifically their cell walls, provide a matrix where the microorganisms can attach; additionally, cell anatomy and cell wall chemistry are expected to influence microbial community structure. *Sphagnum* leaves differ from Amblystegiaceae and other mosses mainly by their unique anatomy, as most of the leaf volume is formed by empty hyaline cells possessing pores. Such cells provide the moss with efficient water conduction and retention, and provide the microorganisms with expanded surface area (inner cell walls), stable hydration and finally more acidic conditions, as the water retention is linked with the retention of acidity^25^.

In the arcto-alpine climate zone, plant species and geographical region were the major determinants of host-specific endophytic bacteria^26^. In contrast, the effect of geographical location on *Sphagnum*-associated microorganisms was reported to be marginal, but vegetation, pH and nutrient richness may potentially determine their distribution^27,28^. Investigations of moss-associated microorganisms of two closely related *Sphagnum* species revealed highly similar diazotrophic and methanotrophic communities, whereas indicator *Sphagnum* mosses in Alpine bogs harboured highly specific diazotrophic bacteria^27,29^. Still, 50% of the Alpine bog microbiome was shared between *Sphagnum*, other bryophytes, vascular plants and lichens and only up to 12% where identified to be *Sphagnum*-specific^30^, suggesting site-specific ‘core microbiomes’ rather than moss-specific microbial communities. The presence and dominance of Acetobacteraceae in the inner tissue of *Sphagnum* sporophyte and in the moss-associated microbial community of the gametophyte indicate that *Sphagnum* species pass on parts of their microbiome from one generation to another^31^. It has been suggested that secondary metabolites produced by the host selects for particular bacterial communities in the *Sphagnum* plants^32^.

While *Sphagnum* has been the subject of several studies on peatland moss-associated prokaryotes, Amblystegiaceae have been largely neglected, despite their major role in peatland formation and predominance in pristine, minerotrophic fens especially in the Polar region^33^. Two comparative studies on nitrogen fixation associated with both *Sphagnum* and brown mosses revealed higher rates for brown mosses and overall large variations between individual moss species^34,35^. Additionally, it was shown that methane oxidizing bacteria are associated with submerged brown mosses, specifically with *Scorpidium scorpioides*^36^, and thus that methanotrophy is not restricted to microbial associations with *Sphagnum*.

There is an apparent knowledge gap regarding integrated studies on the core microbiota and biotic and environmental controls of both Amblystegiaceae- and *Sphagnum*-dominated peatlands. Furthermore, our knowledge of microbial endophytes of Amblystegiaceae and *Sphagnum* mosses is sparse and nothing is known about their role in peatland succession. We hypothesize that the two major peatland types, dominated by the two very different moss taxa Amblystegiaceae and *Sphagnum*, respectively, possess profoundly different microbial communities with few shared species. We designed a large-scale comparative study spanning Amblystegiaceae and *Sphagnum-*dominated peatlands of the Arctic, Subarctic and temperate region. We assessed pH, nutrient levels, moss taxon host-specificity, but also temperature and hydrology as potential controls on the structure and diversity of the peatland microbiota and compared the endophytic and epiphytic microbial communities to the members of the core community.

## Materials and methods

### Study sites and sampling

**Figure 1:**
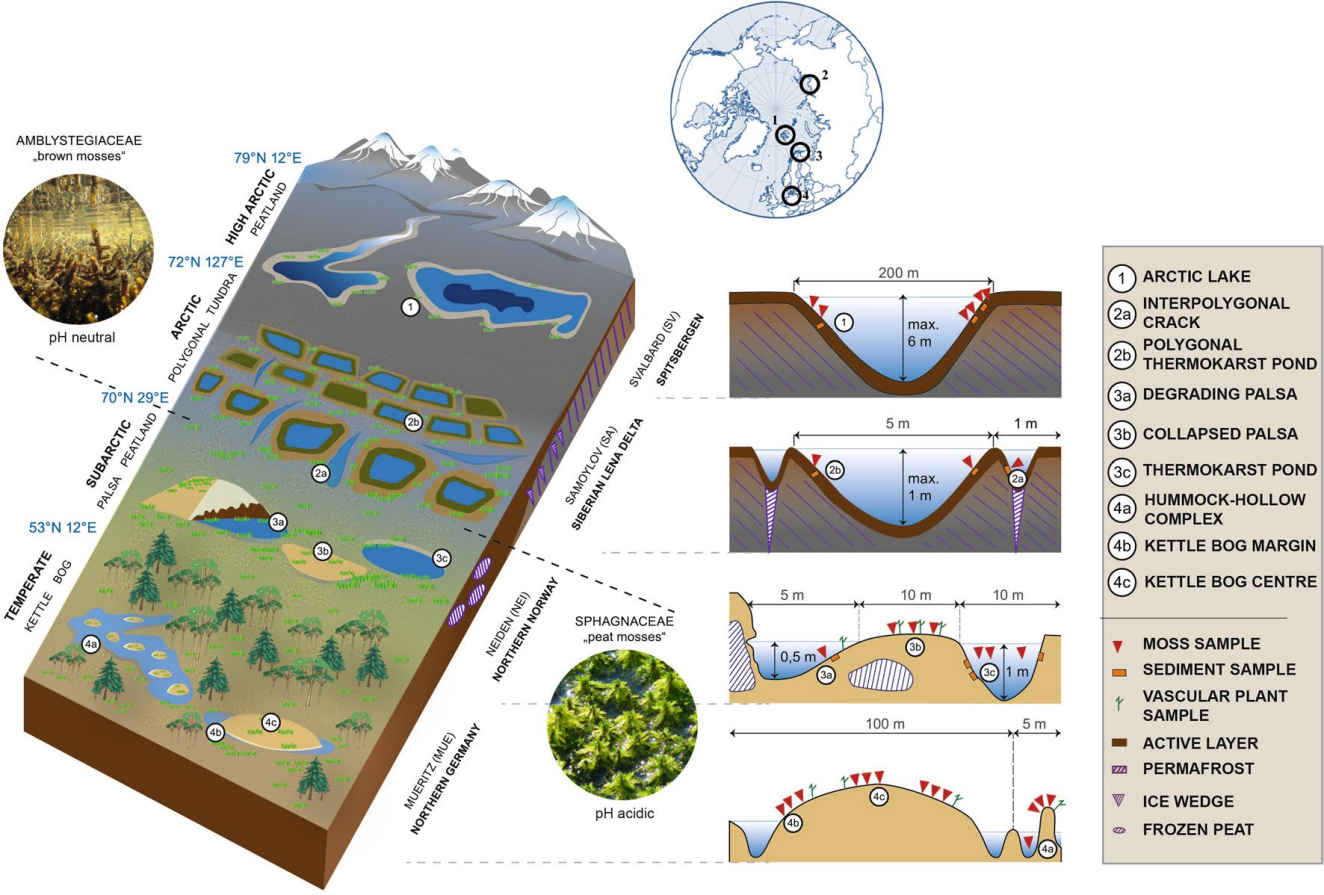
Schematic overview of the investigated areas and the type of samples collected. The geographical location of the sampling sites is depicted above. The different peatland types are illustrated on the left, the sampling scheme is depicted on the right. Subsampling sites are not emphasized. Illustration: Grit Schwalbe, GFZ.

We studied two main ecosystems (Amblystegiaceae- and *Sphagnum*-dominated peatlands), represented by four sites, which are analogous to different stages in the transition from fens to incipient ombrotrophic bogs: 1) High-Arctic lakes with colonies of Amblystegiaceae on Svalbard (SV), Norway; 2) Arctic polygonal tundra with densely growing Amblystegiaceae on Samoylov Island (SA), Lena Delta, Russia; 3) Sub-arctic *Sphagnum* palsa peatlands in Neiden (NEI), Northern Norway, and 4) temperate *Sphagnum* kettle bogs in the Mueritz National Park (MUE), Northern Germany. An overview about the study sites and a simplified sampling scheme is given in Fig. 1, and a detailed overview of the 157 samples and the corresponding sampling sites, biotic (plant species) variables, environmental variables and geographical coordinates is provided in Tables S1A and S1B.

On SV (78.9° N, 11.9° E), we sampled a mixture of submerged Amblystegiaceae from three subsites in the vicinity of Ny-Ålesund: *Bryum pseudotriquetrum in* Twin Water (TW), *Drepanocladus trichophyllus* and *Scorpidium turgescens* in Knudsenheia (KNU) and *Drepanocladus revolvens* and *S. turgescens* in Gludneset (GLU), each in duplicates (sample type 1; all sample types are depicted in Fig. 1). On SA (72.4° N, 126.5° E), we sampled a mixture of submerged *Scorpidium scorpioides* and *Meesia triquetra* from an interpolygonal crack (PC; sample type 2a) and *S. scorpioides* from a polygonal pond (three replicate of plants subsumed to PP; sample type 2b). At both locations, SV and SA, we collected sediment underneath the mosses as references. In NEI (69.7° N; 29.4° E), different successional palsa stages were selected: thermokarst ponds with *Sphagnum riparium* adjacent to degrading palsas (one plant within each of the subsites NEI1, NEI2; sample type 3a), thermokarst ponds with *S. riparium* as remnants of collapsed palsas (one plant within each of the subsites NEI3, NEI4; sample type 3c) and hollows with *Sphagnum lindbergii*, representing old successional stages of previously collapsed palsas (one plant within each of the subsites NEI5, NEI6, NEI7; sample type 3b). From MUE (53.3° N, 13.2° E), three subsites were chosen: Heidbergmoor, a hummock-hollow complex (sample type 4a) with emerged *Sphagnum fallax* (three replicate plants within the subsite called HEI2) and submerged *S. fallax* (one plant within the subsite called HEI1); Klockenbruch, a kettle bog with an oligotrophic, elevated center (sample type 4c) with *Sphagnum magellanicum* (three replicate plants within KLO1) and a meso-oligotrophic, lower margin (sample type 4b) with *S. fallax* (three replicate plants within KLO2); Kiebitzmoor, a formerly drained and rewetted kettle bog (sample type 4c) with *S. magellanicum* (three replicate plants within KIE). In NEI we collected the sedges *Eriophorum* sp. and *Carex* sp. (NEI1, NEI5, NEI6, NEI7) and sediment underneath the mosses (NEI1, NEI2, NEI3, NEI4) as references, with duplicates for each site and reference type. In MUE, *Eriophorum vaginatum* (HEI2, KLO1 and KLO2) and *Carex* sp*.* (KIE) were collected as references with duplicates for each site and plant type. Sampling in all sites was done between June and September 2013.

Peat or moss batches were sampled using gloves and sterile knifes or spoons. Leafs, stem and upper root material of vascular plants were manually extracted from the peat body, washed with sterilized tap water to get rid of microorganisms that came from the surrounding environment and were not attached to the plants, cut and used as a bulk reference sample. Complete moss individuals were sampled and also washed with sterilized tap water for removal of organisms from the surrounding environment prior to storage. All samples were stored at − 80 °C immediately after sampling until further processing except for the samples from Samoylov Island, Lena Delta, that were continuously stored at − 20 °C.

At each site, pore water was retrieved from three depths when possible; slightly above, within and below the moss layer by extracting small samples of pore water with perforated brass tubing as described elsewhere^37^. At the hummock sites, pore water was extracted from the shallowest depth possible. Ten-mL plastic syringes equipped with three-way valves were connected to the brass tubes and used to carefully suck out the pore water. Pore water was transferred to gas-tight 20-mL glass serum vials pretreated with 100 μL 1 M HCl and pre-flushed with N_2_ avoiding air bubbles and stored at 4 °C.

### Pore water analysis (pH, temperature, methane, DOC and O_2_)

Values of pH were measured in the field using a multi parameter probe Multi 350i from WTW (Laboratory and Field Products, Nova Analytics). Air and peat temperatures were measured with a hand-held digital thermometer 2000T (Thermocouple Thermometer, Digitron Instrumentation Ltd, England) equipped with a 50 cm long probe. Headspace methane concentrations were measured by gas chromatography shortly after pore water sampling as described elsewhere^37^. For the determination of DOC values, 20-mL glass vials (Agilent) were flushed with ultrapure water, baked at 550 °C for 2 h, closed with aluminium-sealed PTFE/butyl septa and acidified with 3% HCl Suprapur (VWR). 15 mL of the pore water was filtered with 0.7 µm GF/L filter (Whatman). The samples were sent to ‘Potsdamer Wasser- und Umweltlabor GmbH’ (PWU) for DOC analysis. Pore water O_2_ contents were measured in the field at different depths (above, within and below moss layer, where possible), using an optical oxygen meter (FireStingO_2,_ PyroScience).

### Cell wall analysis (CEC, HC, LLP)

#### Cation exchange capacity (CEC)

Up to 45.0 mg of dry moss samples were sealed into labelled polyamide mesh bags. The bags were submerged in 2 L of 20 mM HCl to soak the moss up and to convert all carboxylic cation-exchange sites to un-dissociated form; free protons were then replaced by repeated thorough wash with distilled water. All the bags were then transferred to 2 L of 0.5 M ammonium acetate and after pH equilibration the ammonium acetate solution was renewed and adjusted to pH 7.0 using NH_4_OH. The bags were repeatedly washed with large amount of distilled water to replace free NH_4_^+^ and dried. The bags were individually immersed to 50 mL of 20 mM HCl and shaken for 15 min to elute cell-wall bound NH_4_^+^ ions. The eluate was sampled and NH_4_^+^ analyzed colorimetrically using Flow Injection Analysis (Foss Tecator AB, Sweden).

#### Holocellulose (HC)

Dry plant samples were ball-milled for 2 min at 30 Hz to fine dust (MM200, Retsch) and about 40.0 mg of the material was washed with 5 mL of 70% acetone in 15-mL Falcon tubes and oven-dried in the tubes at 48 °C. 8 mL of H_2_O, 75 µL of glacial acetic acid and 150 µL of 25% sodium chlorite (NaClO_2_) were added. The tubes were closed shaken and incubated for 1 h in a water bath at 75 °C, being shaken every 10 min. The additions of acetic acid and sodium chlorite and the incubation was repeated three times. Afterwards, samples were cooled and centrifuged at 4000 × *g* for 15 min, supernatant was discarded. 10 mL H_2_O was added, samples were vortexed and centrifuged at 3000 × *g*, supernatant was discarded. This wash step was repeated twice, followed by drying at 70 °C. The residuum is referred to as holocellulose (structural polysaccharides) and expressed in % of dry mass.

#### Lignin and Lignin-like polymers (LLP)

To remove phenolic extractives that can interfere with later spectrophotometric determination of acid-soluble Klason lignin, up to 60.0 mg of milled plant material was shaken with 5 mL of 70% acetone in 15-mL Falcon tubes for 1 h. The tubes were then centrifuged, supernatant discarded and the pellets dried in the tubes at 48 °C. 0.4 mL of 72% H_2_SO_4_ was added to the pellet, the tubes were vortexed and incubated for 1 h at 23 °C, followed by addition of 11.2 mL of H_2_O, vortexing and incubation at 100 °C for 2.5 h. The tubes were then centrifuged at 3000×*g* for 15 min and the supernatant was sampled for dissolved lignin analysis and discarded. The pellet (Klason lignin, acid-insoluble residuum) was washed three times with 10 mL of water, centrifuged, oven-dried at 70 °C and expressed in % of dry mass. Acid-soluble Klason lignin was measured spectrophotometrically at 205 nm (standard mass attenuation coefficient of 110 L g^−1^ cm^−1^ was applied according to Hatfield and Fukushima 2005) and expressed in % of dry mass. Acid-soluble Klason lignin and Klason-lignin were summed to Total Klason lignin (representing lignin-like phenolics in mosses as they lack true lignin).

### Total carbon (TC), total nitrogen (TN), and C:N ratio (C/N)

Plant samples were dried and milled (Pulverisette, Fritsch). About 5.0 mg of sample was weighed in tin boats (Elementar). TC and TN contents were determined as double measurements with a carbon, nitrogen and sulfur (CNS) analyser (Elementar Vario EL III). For determining C/N, quotients of TC and TN were calculated.

### Separation of loosely (epiphytic) and closely associated (putative endophytic) microorganisms

Between 2.2 and 5.3 g of the moss material pre-treated as described above was thawed and amended with extraction buffer containing ultrapure DEPC water (AppliChem), 0.85% NaCl (Merck), and 0.01% Tween20 (AppliChem) in a ratio 2:1 (weight percent), a modification of a previously published protocol^38^. The mixture was shaken horizontally for 1 h at 4 °C prior to ultrasonication (Bandelin Sonoplus HD3100) with pulsation for 2 min (1 s off, 2 s on) at 0.45 W/mL^39^. Extraction buffer containing the epiphytes was filtered through a 0.2 µm cellulose filter (Sartorius Stedium). The remaining moss was surface-sterilized with 0.15% NaOCl (Roth) for 1 min, and rinsed 7 times with DEPC water according to a modified protocol^40^. Filters and sterilized mosses were ground to powder under sterile conditions with liquid nitrogen, transferred to lysis tubes and stored at − 20 °C until DNA extraction. For each moss sample, one filter with wash-off (epiphytes) and two technical replicates of the surface-sterilized moss (putative endophytes) were used for DNA extraction and sequencing (below).

### DNA extraction and sequencing

Genomic DNA was extracted from 0.4–0.8 g of each of the surface treated mosses, the filters containing the wash-off as described above, and from about 0.4–0.8 g untreated sedges and sediment samples as references for the moss samples following the CTAB/phenol–chloroform-based method of^41^. DNA concentrations were quantified with a Nanophotometer P360 (Implen GmbH, München, DE) and a Qubit 2.0 Fluorometer (Thermo Fisher Scientific, Darmstadt, Germany) according to the manufacturer’s protocols. Bacterial 16S rRNA genes were amplified with the primer combination S-D-Bact-0341-a-S-17 and S-D-Bact-0785-a-A-21^42^ and archaeal 16S rRNA genes were amplified with the primer combination S-D-Arch-0349-a-S-17 and S-D-Arch-0786-a-A-20^43^. The primers were labelled with different combinations of barcodes listed together with primer sequences in table S1B. The PCR mix contained 1 × PCR buffer (Tris·Cl, KCl, (NH_4_)_2_SO_4_, 15 mM MgCl_2_; pH 8.7) (QIAGEN, Hilden, Germany), 0.5 µM of each primer (Biomers, Ulm, Germany), 0.2 mM of each deoxynucleoside (Thermo Fisher Scientific, Darmstadt, Germany), and 0.025 U µl^-1^ hot start polymerase (QIAGEN, Hilden, Germany). The thermocycler conditions were 95 °C for 5 min (denaturation), followed by 40 cycles of 95 °C for 1 min (denaturation), 56 °C for 45 s (annealing) and 72 °C for 1 min and 30 s (elongation), concluded with a final elongation step at 72 °C for 10 min. PCR products were purified with a Hi Yield Gel/PCR DNA fragment extraction kit (Süd-Laborbedarf, Gauting, Germany) according to the manufacturer’s protocol. PCR products of three individual runs per sample were combined. For sequencing, PCR products of different samples were pooled in equimolar concentrations and compressed to a final volume of 10 µL with a concentration of 200 ng/µL in a vacuum centrifuge Concentrator Plus (Eppendorf, Hamburg, Germany). The library preparation and sequencing was performed on an Illumina MiSeq sequencer by the company GATC (Konstanz, Germany) according to their standard protocols. In short, the library was prepared with the MiSeq Reagent Kit V3 for 2 × 300 bp paired-end reads. To account for the low-diversity amplicon sampling, we used 15% PhiX control v3 library.

### Sequence analyses and bioinformatics

Raw data was demultiplexed using CutAdapt^44^; e 0.1; –trim-n; no error in barcodes allowed. Paired-reads were merged using PEAR^45^ (Q25; p 10^-4; v20) and sequence orientation was standardized using own scripts. Low quality sequences were filtered and trimmed using Trimmomatic^46^ (LEADING:25; TRAILING:25; SLIDINGWINDOW:5:25; MINLEN:200). Chimeras were removed according to the QIIME SOP^47^. Finally, reads were clustered into Operational Taxonomic Units (OTUs) using QIIME’s *pick_open_reference.py* script with a cutoff value of 97%^47^. Representative sequences of the clusters were annotated with usearch utilizing the curated Greengenes 13.8 taxonomy database^48^. OTUs assigned to chloroplasts, bacterial OTUs within archaeal samples and vice versa, and OTUs with a small, sample-wise relative abundance (< 0.01%) were filtered before further exploration.

### Statistical analyses

Differences in microbial community composition between the sites were obtained by calculating the inverse Simpson index and counting the number of OTUs, as measures of the OTU diversity and richness, respectively. Bubbleplots for Figs. 5 and 6 were generated using the package ‘ggplot2’ (version 2.2.0) within the statistical software R (version 3.2.2)^49^. Correlation matrices of samples (16S rRNA gene datasets of either bacteria or archaea) were generated using the R function ‘cor’, specifying the Spearman rank correlation coefficient. Hierarchical clustering of the samples based on the correlation matrices to generate dendrograms were calculated using the method ‘agnes’ within the R package ‘cluster’, with default settings. All heatmaps were created using the R package ‘heatmap3’ (version 0.3.3). The inverse Simpson index diversity estimates for bacteria were calculated using the R package ‘asbio’ (version 1.6-5). Pairwise t-tests were used for environmental variables and carried out using the R function ‘pairwise.t.test’. Pairwise Mann–Whitney-Wilcoxon tests were used for diversity indices and carried out using the R function ‘pairwise.wilcox.test’. Canonical correspondence analysis (CCA) was carried out to quantify the explanatory power of biotic and environmental variables with respect to the microbial ecology of the peatlands (package: vegan (version 2.2.1)). Correspondence analysis (CA) was carried out as described in^50^ and plotted using ‘ggplot2’. Eight variables; cation exchange capacity, lignin-like polymers, hemicellulose, total nitrogen, total carbon and C:N ratio, DOC, oxygen and water content were removed from the initial full model due to lacking observations for between 23 and 45% of the samples. We constrained the variation in the microbial communities to the remaining variables; (1) sites (SV, SA, MUE and NEI), (2) subsite (e.g., KIE1), (3) plant species or reference sediment, (4) location above or below water table, (5) washed and surface-sterilized moss plant (putative endophytes) or wash-off (epiphytes) (6) pH, (7) methane concentration in pore water, and 8) temperature. In order to estimate and account for the spatial autocorrelation that the sites (1) and subsite (2) variables represent, we introduced partial CCA. Running the model without (1) and (2) we observed that the constrained inertia was reduced from 72% of total inertia to 40%. Subsequent analysis of variance inflation factors showed that no remaining variables were redundant. Core communities were calculated with a restrictive 66% threshold, meaning that an OTU has to be present in 80 out of 122 samples and in both system types (Amblystegiaceae and *Sphagnum*) for it to be considered part of the core microbiota. Moss system core communities (Amblystegiaceae or *Sphagnum*) were calculated with the same threshold, 66%. Moss species communities were calculated with a more restrictive threshold of 75%^30^.

## Results

### Peatland characteristics

**Figure 2:**
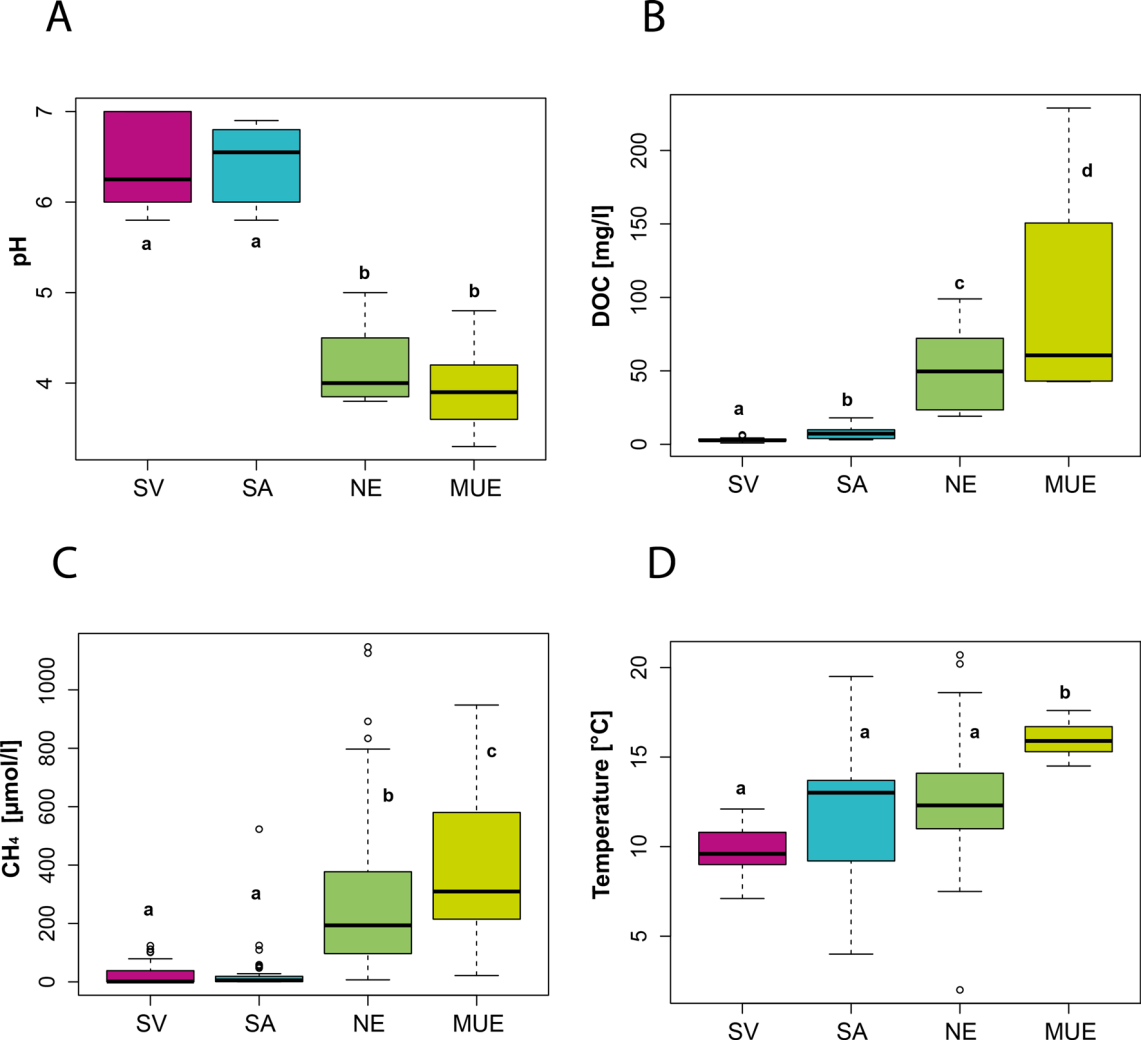
Box plots showing the measurements of selected environmental variables. (**A**) pH, (**B**) dissolved organic carbon (DOC), (**C**) methane and (**D**) temperature of all subsites in Svalbard (SV, magenta), Samoylov (SA, blue), Neiden (NEI, dark green) and Mueritz (MUE, light green). Pairwise t-tests suggest that samples with different letters show a significant (p < 0.05) difference in the mean value between each other.

The sites Svalbard (SV) and Samoylov (SA) contained only mosses of the family Amblystegiaceae and represented minerotrophic fens at the earliest stages of peat formation (‘terrestrialisation’), with sub-neutral to neutral pH values ranging from 5.8–7.0. The sites Neiden (NEI) and Mueritz (MUE), dominated by the genus *Sphagnum*, represented later stages of peat formation, (´paludification´) spanning minerotrophic and nearly ombrotrophic bogs with acidic pH values ranging from 3.3–5.0, thus significantly lower than in SV and SA (Fig. 2A). DOC values were significantly higher in *Sphagnum* than in Amblystegiaceae peatlands, with the highest concentrations observed in MUE (42.7–229 mg/L) and lowest in SV (0.9–6.4 mg/L) (Fig. 2B). Also methane concentrations were significantly higher in the *Sphagnum* than in Amblystegiaceae ecosystems, with the highest range of concentrations in MUE (21.8–948 µM) and the lowest in SV (0–124 µM) (Fig. 2C). The mean soil temperature at the time of sampling was highest in MUE (16.0 °C, range 14.5–17.6), followed by SA (13.0 °C, range 4.0–19.5), NEI (12.6 °C, range 2.0–20.7 °C) and SV (9.8 °C, range 7.1–12.1 °C) (Fig. 2D).

### Diversity and structure of natural peatland microbial communities

We obtained between 2510 and 289,604 sequences (average of 78,933, median of 68,070 and std.dev. of 60,092) for the 122 bacterial datasets and between 536 and 83,642 (average 12,626, median of 4892 and std.dev. of 17,424) sequences for the 86 archaeal datasets. Consequently, of the 157 samples collected we were unable to generate 16S rRNA gene amplicon libraries from 35 samples with bacterial primers and 71 samples with archaeal primers either because PCR gave no product or sequencing failed. There was a significantly higher bacterial diversity and OTU richness in the moss and reference samples from Amblystegiaceae*-*dominated SV and SA sites compared to the *Sphagnum-*dominated NEI and MUE sites (Fig. S1). Interestingly, the microbial communities in the Amblystegiaceae sites displayed the same level of diversity, independent of the geographical location. This also applied to the *Sphagnum* sites. Unlike the bacterial diversity, the archaeal diversity was similar between the Amblystegiaceae and *Sphagnum* peatlands with overall little differences between mosses, sediments and vascular plants (Fig. S2). An exception was that the sediments in the Amblystegiaceae sites displayed slightly higher archaeal richness than the other sites.

To identify the association between moss taxa, abiotic environmental variables and the bacterial moss microbiota, we performed a canonical correspondence analysis (CCA). Using variance partitioning we quantified the contribution of the variables to the explanation of total inertia in the following order from most to least important: (1) plant species and reference sediment: 19.7% (p. value < 0.001), (2) temperature: 4.8% (p. value < 0.001), (3) putative endophytes or epiphytes: 4.6% (p. value < 0.001), (4) methane concentration in pore water: 4.1% (p. value < 0.001), (5) pH: 3.3% (p. value < 0.001) (6) location above or below water table: 3.2% (p. value < 0.001). Repeating the procedure with Hellinger transformed data to control for large effects of low abundant OTUs we observed the same patterns at highly similar total and constrained inertia, suggesting that the impact of rare OTUs on CCA ordination was minor. Initially, our model included sites and subsites in addition to the six above mentioned variables, together accounting for 32% of the differences between the microbial communities (see materials and methods). As site effects and plant species effects correlate, it is likely that by removing site effects we have substantially underestimated the plant species influence. The removed fraction of the inertia contained in the site variables we consider the ‘environment’, a mix of abiotic and biotic variables that cannot be studied in isolation with our dataset.

**Figure 3:**
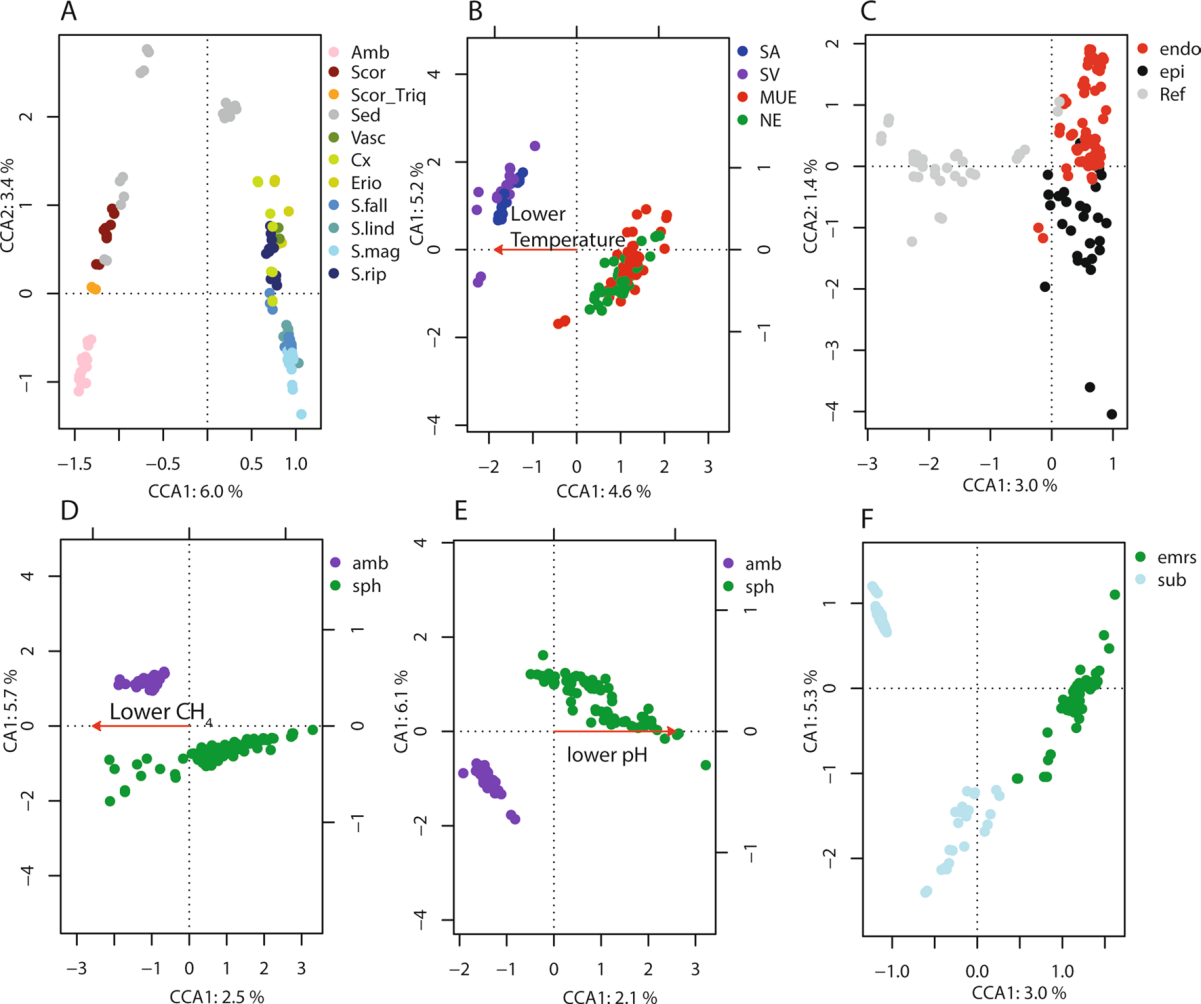
Canonical correspondence analysis of moss-associated bacterial OTUs. The axes represent the first and second CCA dimension in the case of categorical variables with more than two factors (**A**,**C**). In the case of 2 factors or continuous variables (**B**,**D**,**E**,**F**) the first CA dimension is showed on the Y-axis, while the CCA dimension is showed on the X-axis. (**A**) Constrained by plant species and reference sediment; Amb: Amblystegiaceae mix; Cx: Carex; Erio: Eriophorum; S.fall: *Sphagnum fallax*; S.lind: *Sphagnum lindbergii*; S.mag: *Sphagnum magellanicum*; S.rip: *Sphagnum riparium*; Scor: *Scorpidium scorpioides*; Scor_Triq: Mix of *Scorpidium scorpioides* and *Meesia triquetra*; Sed: Sediment; Vasc; Mix of Vascular plants. (**B**) Constrained by location above or below the water table; emrs: Above the water table; sub: below the water table. (**C**) Constrained by being from the washed moss plant (putative endophytic: endo), wash-off (putative epiphytic: epi) or from reference sample (Ref). (**D**) Constrained by pH, samples coloured by System; amb: Amblystegiaceae; sph: *Sphagnum*. (**E**) Constrained by temperature, samples coloured by Area; *MUE* Mueritz; *NEI* Neiden; *SA* Samoylov; *SV* Svalbard. (**F**) Constrained by CH_4_ concentration in pore water, samples colored by System; *amb* Amblystegiaceae; *sph* *Sphagnum*.

Due to its complexity, we were unable to visualize the major gradients in the dataset using a single CCA plot. Thus, we plotted the constraints of the final model above separately (Fig. 3). The plots show that substantial parts of the bacterial communities correlate with the moss or vascular plant species (Fig. 3A) and by being submerged in water or above (Fig. 3F). At one instance we saw that the water table had a stronger impact on the microbiota than the plant species; the *S. fallax* samples closest to the *S. riparium* in Fig. 3A were submerged, as were all the *S. riparium* samples. There were also consistent differences between endophytic and epiphytic communities (Fig. 3C). Furthermore, in line with the differences in pH and temperature, we observed clear differences between the Amblystegiaceae and *Sphagnum* bacterial communities (Fig. 3B,E), while the effects of altered methane concentrations on the microbial communities were similar in Amblystegiaceae and *Sphagnum* ecosystems (Fig. 3D). The CCA explained approximately 40% of the variance in the dataset. The small explained variance was to a large extent due to the removal of area and subsite variables which was not considered explanatory variables.

**Figure 4:**
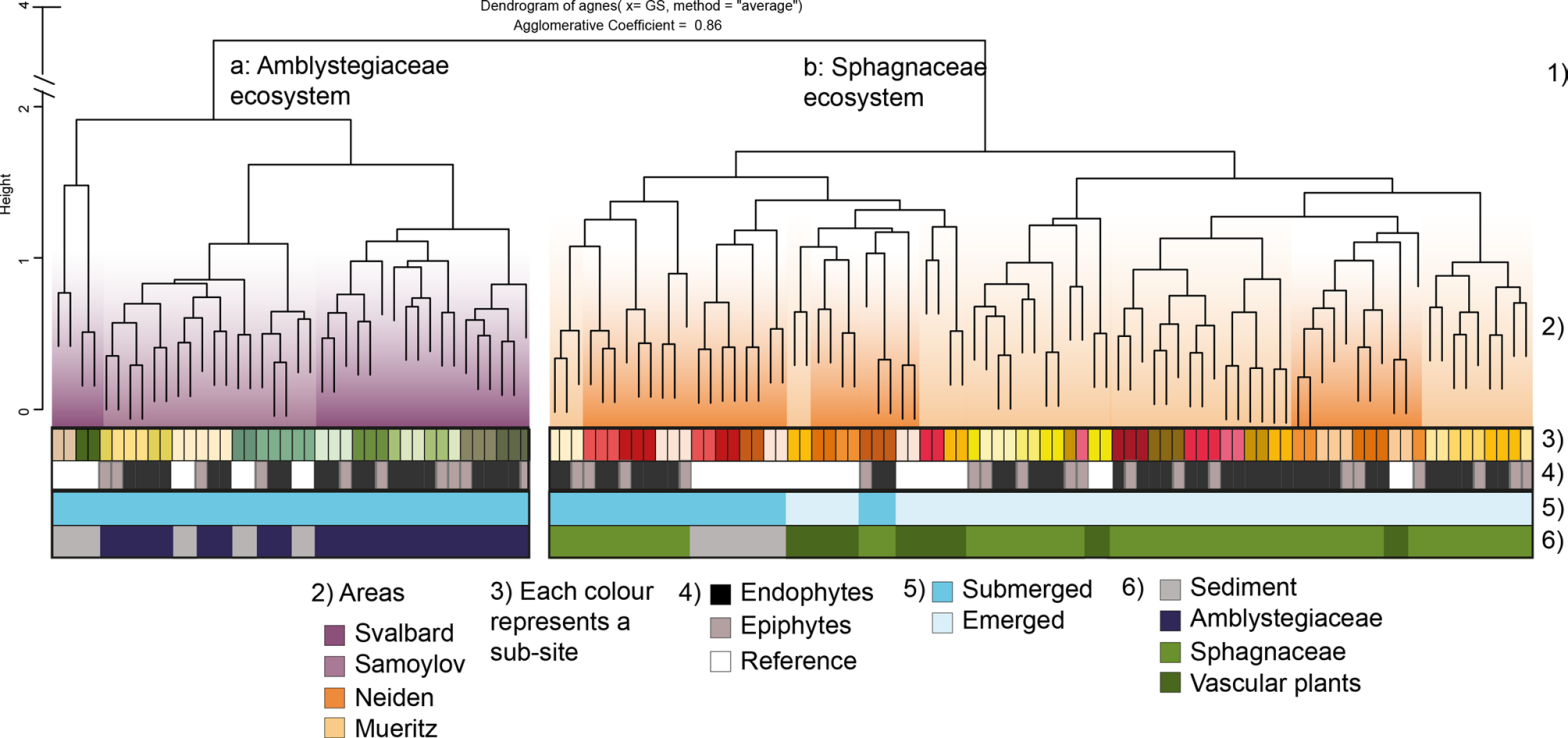
Dendrogram showing the clustering of bacterial communities (OTU at 97% sequence similarity) in relation to the environmental and biotic characteristics of the sites. Each tip of the dendrogram corresponds to the community profile of a moss, vascular plant or sediment sample. All possible pairwise spearman correlation factors were calculated from the community profiles and the resulting distance matrix used to cluster the samples applying the agnes hierarchical clustering algorithm. Numbers refer to different levels of clustering. The first level of clustering, (1), shows that the samples from ecosystems dominated by Amblystegiaceae and the samples from ecosystems dominated by *Sphagnaceae* end up in two different clusters. The second level of clustering, (2), shows that the majority of samples from the same sites cluster together, but that exceptions related to hydrology and reference samples occur. Level (3) shows that bacterial communities from the same subsite almost exclusively cluster together. Level (4) show that the putative endophyte amplicon libraries from the washed moss plant almost always cluster with the wash-off library containing the putative epiphytic library from the same moss plant. Level (5) show a clear microbial community separation by the hydrology of the site. Level (6) show some clustering based on whether the community is from a vascular plant, sediment or moss.

To allow an evaluation of this area and site-dependent structure of the microbial communities we constructed a Spearman correlation based dendrogram of the OTU profiles, along with some of the categorical variables. The analysis revealed a very high level of agglomerative clustering in the dataset (0.86), particularly considering the large size of the dataset. The resulting dendrogram confirmed some of the previously observed patterns, such as the differences associated with dominating moss vegetation (Amblystegiaceae or *Sphagnum*) and hydrology (Fig. 4). However, it also revealed additional data structures. Starting from the top of Fig. 4, we can see the bacterial communities split by (1) the overall system and moss type, Amblystegiaceae or *Sphagnum-*dominated. (2) With few exceptions the bacterial communities within the two systems split by areas. (3) In almost all cases, the communities from the same subsites clustered together. (4) Within each subsite, the epiphytic communities clustered separately from the endophytic communities, but consistently the endophytic and epiphytic libraries from the same plant clustered together. (5) The submerged NEI and MUE moss communities clustered together. (6) Within the *Sphagnum* system, most of the vascular plant communities clustered together with the sediment and submerged moss communities.

### Amblystegiaceae- and *Sphagnum*-associated microbial taxa

**Figure 5:**
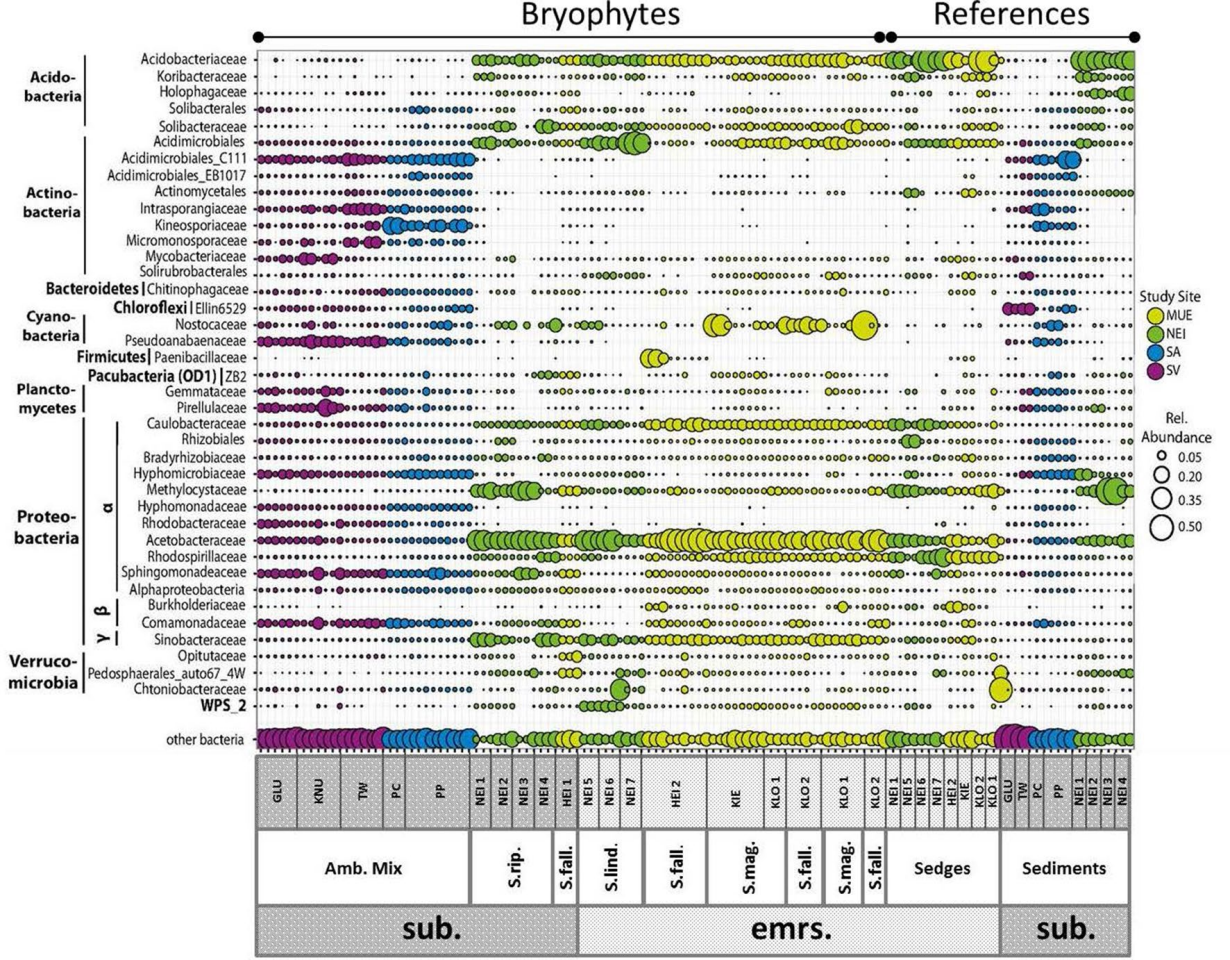
Overview of the relative abundances of bacterial families making up more than 0.5% of the total bacterial sequences in one or more 16S rRNA gene libraries. The sizes of the circles correspond to the relative abundances of the families. The colors indicate which region the samples originate from. MUE: Mueritz, Northern Germany (light green); *NEI* Neiden, Northern Norway (dark green); *SA* Samoylov, Russia (blue); *SV* Svalbard, Norway (violet). The samples are sorted by ecosystem types and latitude from left to right. sub. = submerged. emrs. = emerged/above the water table. Amb. Mix. = a mix of *Amblystegiaceae*. S. rip. = *Sphagnum riparium*. S. fall. = *Sphagnum fallax.* S. mag = *Sphagnum magellanicum*. S. Lind. = *Sphagnum lindbergii*.

To identify which bacterial and archaeal groups accounted for the majority of microbial community variation we studied the communities at family level. Within bacteria, we observed an evenly high abundance of families in the Amblystegiaceae moss microbiota; Acidimicrobiales_C111, Pseudoanabaenaceae, Hyphomicrobiaceae, Sphingomonadaceae and Comamonadaceae (Fig. 5).

In contrast, only two bacterial families dominated the *Sphagnum* moss microbiota; Acetobacteraceae and Acidobacteriaceae*.* Sphingomonadaceae was the only family present at similar relative abundances in the Amblystegiaceae and *Sphagnum* systems. To identify the nature of these large differences we studied the OTU composition of Acetobacteraceae in more detail (Fig. S3). The relative abundance of Acetobacteraceae was higher in *Sphagnum* than in Amblystegiaceae systems and the majority of Acetobacteraceae OTUs were present only in *Sphagnum*. However, some OTUs were only present in Amblystegiaceae, while a handful of OTUs were present in both the Amblystegiaceae and *Sphagnum* peatlands. To identify whether this distinctly different OTU composition with few overlaps was a general pattern, we repeated the analysis for other major bacterial taxa. The same pattern was observed for Acidobacteria (Fig. S4), Acidimicrobiales (Fig. S5), and Cyanobacteria (Fig. S6). This points towards distinct bacterial communities of *Sphagnum-* and Amblystegiaceae-dominated peatlands, while only individual OTUs occurred in both peatland types. Among the community of methane oxidizing bacteria (MOB), *Methylocystis* was most abundant (Fig. S7). Unlike most bacterial taxa, *Methylocystis* occurred in almost all of the sites but its relative abundance varied and correlated positively with the amount of methane in the pore water. In addition to *Methylocystis*, the MOB community contained members of within *Methylomonas*. In contrast to *Methylocystis,* OTUs affiliated with *Methylomonas* occurred primarily in *Sphagnum* sites, preferentially under submerged conditions. Finally, *Methyloferula-*associated OTUs were also detected among MOB but at low relative abundances. These OTUs were restricted to emerged *Sphagnum* sites. The fasta files of methanotrophic OTUs in Fig. S7 is provided as additional supplement (S_methanotrophs_fasta). The complete OTU table for bacteria is provided as supplementary information (Supplement_Bacteria_OTU_table).

**Figure 6:**
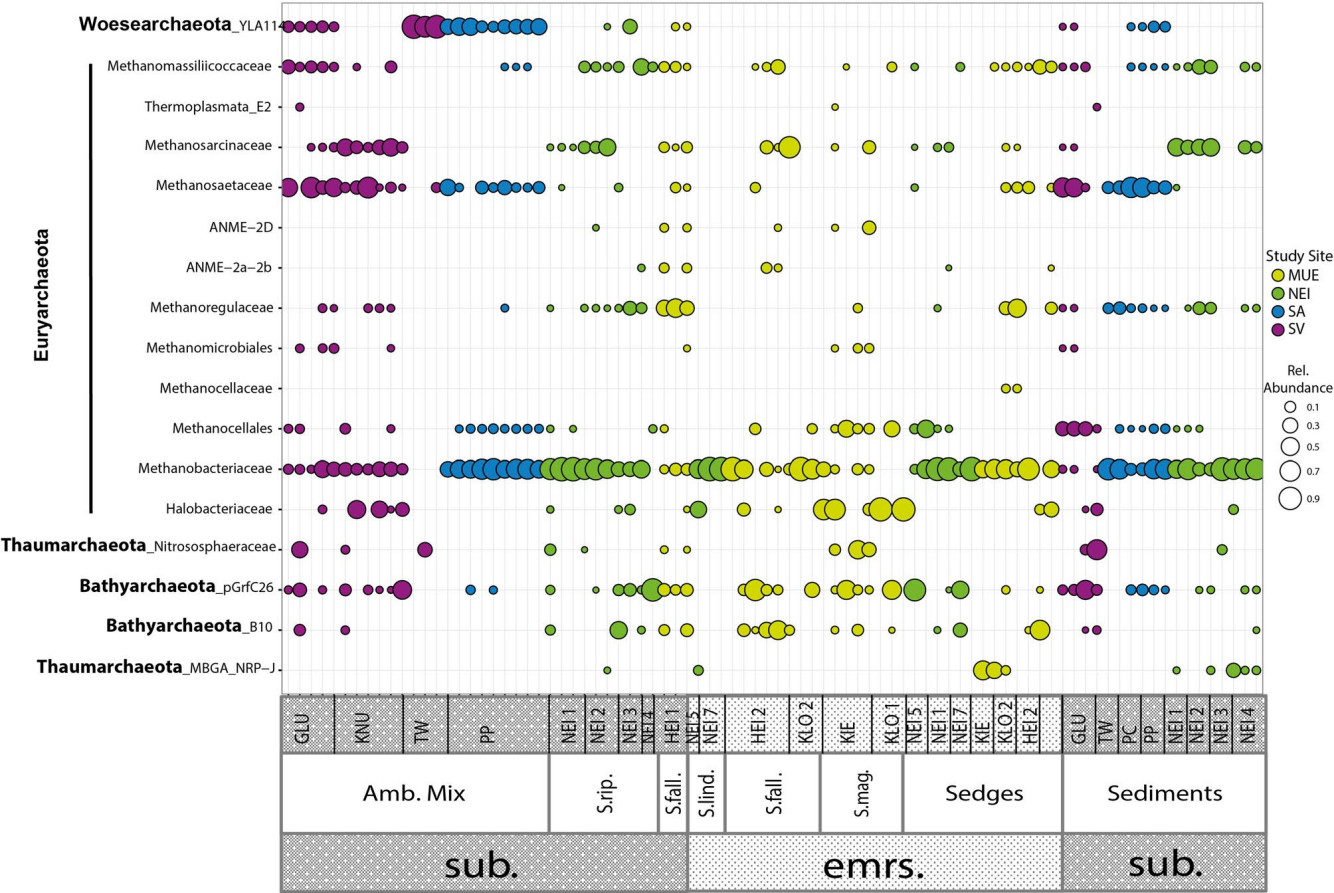
Overview of the relative abundances of archaeal families making up more than 0.5% of the total archaeal sequences in one or more 16S rRNA gene libraries. The sizes of the circles correspond to the relative abundances of the families. The colors indicate which region the samples originate from. *MUE* Mueritz national park; *NEI* Neiden, Northern Norway; *SA* Samoylov, Siberia; *SV* Svalbard, Norway. The samples are sorted by ecosystem types and latitude from left to right. sub. = submerged. emrs. = emerged/above the water table. Amb. Mix. = a mix of *Amblystegiaceae*. S. rip. = *Sphagnum riparium*. S. fall. = *Sphagnum fallax.* S. mag = *Sphagnum magellanicum*. S. lind. = *Sphagnum lindbergii*.

Clustering of the archaeal communities did not reveal any hierarchical clustering patterns related to sample origin (Fig. S8), as was observed for bacteria. The OTUs within the phylum Euryarchaeota, the majority of which belonged to families/genera of methanogenic archaea, dominated the archaeal communities (Fig. 6).

The most abundant OTU belonged to the hydrogenotrophic methanogenic family Methanobacteriaceae. This OTU was present in almost all the samples of both Amblystegiaceae and *Sphagnum* ecosystems (Fig. S9). Furthermore, Methanomassiliicoccaceae, Methanocellales, and Methanosarcinaceae were widespread while Methanosaetaceae occurred mainly in the Amblystegiaceae sites. Outside the phylum of Euryarchaeota, the Bathyarchaeota were abundant throughout most of the sites while Woesearchaeota mainly occurred in the Amblystegiaceae sites. The complete OTU table for archaea is provided as supplementary information (Supplement_Archaea_OTU_table).

### The core and moss-associated microbiota

Only about 0.4% of all bacterial OTUs identified (49 out of 13,799) were observed in both *Sphagnum* and Amblystegiaceae ecosystems and were designated as the core microbiome (see materials and methods for definition of core microbiome). The majority of these OTUs belonged to Acetobacteraceae and Acidobacteriaceae, the dominating bacterial families of the *Sphagnum* microbiota. These 49 OTUs (52 if only considering mosses) contributed 1 – 9% of the total OTU abundance in Amblystegiaceae ecosystem and 12 – 65% in *Sphagnum* ecosystem, showing that the OTUs present in both systems are among the most abundant OTUs in *Sphagnum* sites (Table S2A). Further, we addressed whether the core microbiome of all mosses was similar in size to the individual bacterial core microbiome of Amblystegiaceae and *Sphagnum* mosses (moss system core communities), respectively. We found that by applying the same threshold as for the total core microbiome (TCM), the Amblystegiaceae core microbiome (ACM) was 348 OTUs, while the *Sphagnum* core microbiome (SCM) was 142 OTUs (Table S2A). Out of these, 20 were shared between TCM and ACM, while 46 were shared between TCM and SCM. To identify whether specific moss types shared larger core microbiomes, we calculated the moss species communities of the Amblystegiaceae mosses from Svalbard, the Amblystegiaceae mosses of Samoylov (only *Scorpidium*), *Sphagnum riparium*, *S. fallax*, *S. lindbergii* and *S. magellanicum*, respectively (Table S2B). This showed that the individual core microbiomes are in a similar size range as for the broader core microbiomes at 295, 548, 126, 132, 252 and 154 OTUs, respectively. Calculating the intersects of these core microbiomes we found that the *Sphagnum* mosses share a larger proportion of their core microbiomes with each other than with the Amblystegiaceae mosses (Table S2C). Interestingly, *Scorpidium* mosses shared more OTUs with the *Sphagnum* species than Amblystegiaceae mosses from Svalbard shared with *Sphagnum* mosses. The Svalbard and Samoylov mosses shared the highest number of OTUs, reflecting the larger overall number of OTUs associated to these mosses and their larger core microbiomes. Consistently for all core microbiomes calculated, the relatively few OTUs compared to the total number of OTUs identified accounted for a large proportion of the relative abundance in the microbial communities.

**Figure 7:**
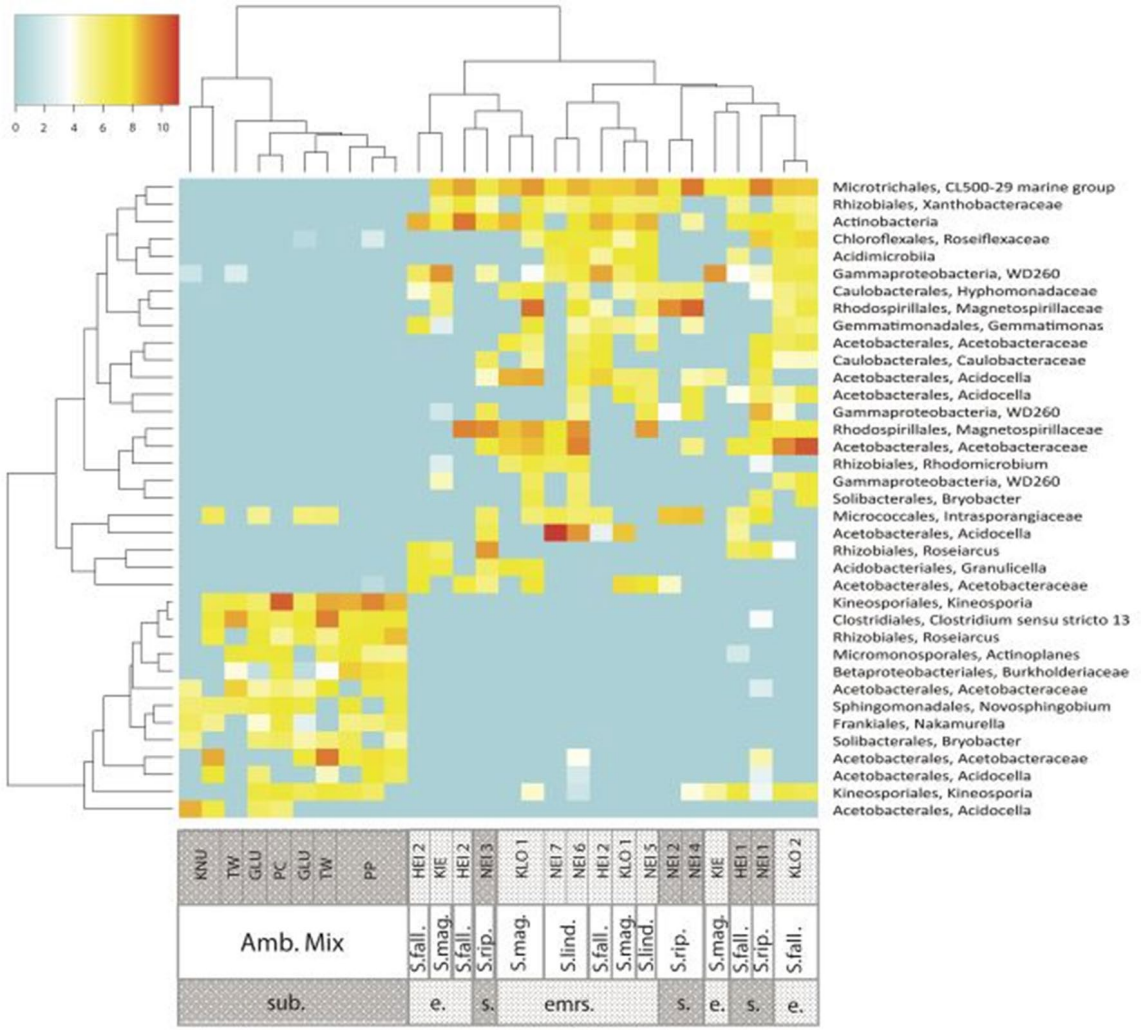
Heatmap displaying the most abundant OTUs present at significantly higher abundance in putative endophytic than epiphytic libraries of the same sample. Chi-square contingency table tests were applied, where the p-values were calculated for Monte Carlo simulations with 5,000 replicates. The significance threshold was set at 0.001. Of the OTUs present at significantly higher abundance in the putative endophytic than epiphytic libraries, only OTUs at a higher than 0.5% relative abundance (average of the two endophytic libraries of each sample) in four or more samples were plotted in the heat map. The color intensity corresponds to the binary logarithm of the average relative abundance of the OTU in the two endophytic libraries multiplied by 100,000. Pearson correlation was used as the basis for the hierarchical clustering of samples and OTUs in the heatmap. sub. = submerged. emrs. = emerged/above the water table. Amb. Mix. = a mix of *Amblystegiaceae*. S. rip. = *Sphagnum riparium*. S. fall. = *Sphagnum fallax.* S. mag = *Sphagnum magellanicum*. S. Lind. = *Sphagnum lindbergii*. Bacterial communities of brown moss samples from Twin water (TW), Gludneset (GLU) and Knudsenheia (KNU) in Svalbard and from polygonal crack (PC) and polygonal pond (PP) in Samoylov. Bacterial communities of *Sphagnum* samples from Klockenbruch (KLO), Kiebitzmoor (KIE), Heidbergmoor (HEI) in Germany, and Neiden (NEI) in Northern Norway.

Next we sought to identify the dominant endophytic communities of Amblystegiaceae and *Sphagnum* mosses. Thus, we plotted the most abundant OTUs of significantly higher abundance in endophytic than epiphytic communities. This showed that almost none of the most abundant putative endophytes (washed and surface sterilized moss samples) of Amblystegiaceae were shared with *Sphagnum* (Fig. 7).

The taxonomic assignment and list of fasta files for the putative endophytes is provided as supplementary material (S_endophytes_taxonomy; S_endophytes_fasta). The *Sphagnum* endophytes belonged to several families within Proteobacteria and Acidobacteria, while the Amblystegiaceae endophytes belonged to Actinobacteria, Proteobacteria, Chloroflexi, Firmicutes and Gemmatimonadetes. Interestingly, of the 24 most abundant *Sphagnum* endophyte OTUs, 19 were observed in the total core microbiome. Thus these are primarily epiphytes of Amblystegiaceae.

## Discussion

We studied the relationship between the moss-associated microbial communities and their respective moss taxa and physico-chemical environments in four sites, in which two were analogous to early (mosses of the family Amblystegiaceae) and two to later (mosses of the genus *Sphagnum*) successional stages of natural northern peatlands. We identified both biotic and abiotic controls on the microbial community structure and distinctly different communities in the Amblystegiaceae and *Sphagnum* mosses. The core community consisted of few but highly abundant members of the microbial community, of which many were endophytes of *Sphagnum*.

### Biotic and environmental drivers of moss-associated bacterial communities

Our study approach allowed ranking the influence of individual variables on microbial community structure relative to the combined dataset. Given that the sum of individual variables reduced the explanatory power by almost 50% compared to the combined dataset that included also area and subsites, and also considering the hierarchical clustering in the bacterial dendrogram, we conclude that a large set of biotic and environmental variables correlate with the ecosystem type, *Sphagnum-* or Amblystegiaceae-dominated peatlands, and that the sum of these exert a major influence on the bacterial community structure. This is in accordance with other studies where soil bacterial communities were reported to structure according to ecosystems^51^ and characteristic microbial communities which evolved in contrasting peatland ecosystems that differed mainly in vegetation, water chemistry and hydrology^52,53^. *Sphagnum* versus brown moss cover was also suggested as the major factor determining diversity and assemblages of testate amoebae^54^. Not only did the peatland ecosystem shape bacterial community structure, but also species richness and diversity, which was significantly higher in sub-neutral Amblystegiaceae compared to acidic *Sphagnum* peatlands, corresponding to findings from neutral compared to acidic soil environments^51,55^. Presumably, most bacteria lack substantial mechanisms to regulate their intracellular pH close to neutral when exposed to low extracellular pH and thus are not able to survive under acidic conditions^56^. Contrarily, most bacteria adapted to low pH can survive at neutral pH, possibly explaining the occurrence of Acidobacteriaceae and Acetobacteraceae in all samples studied, but in much higher abundances in sites with low pH. Acetobacteraceae and Acidobacteriaceae were previously reported to be among the dominant *Sphagnum*-associated microbial groups^31,57,58^ and generally thrive in low pH habitats^59,60^.

Among the individual variables, the specific plant taxa explained four to five times more of the observed inertia than any of the other controlling variables such as temperature, hydrology, and pH. This essentially shows that the different plants harbor their own microbiome which is similar to that of closely related plants. Differences in bacterial communities between different *Sphagnum* species were shown previously^27,31,32^. Our findings extend this knowledge on the influence of the host plant on bacterial community structure to other *Sphagnum* species such as *S. riparium* and *S. lindbergii*, to *Scorpidium scorpioides,* a member of the Amblystegiaceae, and also to some vascular peatland plants like *Carex* and *Eriophorum*. It further supports that although the sediment microbiome does correspond with the different dominant species, it is distinct from that associated with plants as indicated before^61^.

The relationship between temperature and microbial community structure accounted for only 4.8% of inertia in the partial CCA although the investigated mosses were located in different climatic zones. Here, temperature is based on a point measurement and thus not necessarily expected to be representative of growing season average temperatures. Still, a poor influence of temperature on bacterial assemblages both on short and long time-scales is in line with other studies^62,63^. Hydrology was identified as another variable that affects moss-associated bacterial communities in natural peatlands. In some instances hydrology was more important than the influence of the host plant; emerged and submerged *S. fallax* sampled from the same subsite (HEI) carried different microbial communities while the microbiota of submerged *S. fallax* (Mueritz, Germany) was more similar to submerged *S. riparium* (Neiden, Norway) than *S. fallax* growing above the water table. This confirms a strong influence of hydrology on host moss morphology and physiology^64–66^ and the associated microbial communities. The influence of the water table on moss-associated microbial community structure and activity was already reported for *Sphagnum*^20,24,67^ but here we extend it to a larger spatial and environmental scale including Amblystegiaceae. *Sphagnum* and Amblystegiaceae-associated archaea were dominated by methanogenic Euryarchaeota, Bathyarchaeota and Woesearchaeota. This supports the hypothesis that Woesearchaeota occur in methanogenic environments, possibly as syntrophic partners with methanogens^68^. The role of Bathyarchaeota is unclear, but its presence corresponds to its former observations in peatlands^69^. There was some site-dependent clustering of the archaeal communities, e.g. *Methanosaeta* was only found in association with the neutral Amblystegiaceae mosses and associated sediments which is consistent with the biogeography of Methanosaetaceae^70^. The most abundant methanogen, belonging to *Methanobacterium*, was present in all sites and samples, which is also in accordance with the biogeography of *Methanobacterium* being among the most abundant methanogenic taxa both in pH neutral and low pH soils^70^. Similar to the community of methanogens, also the most abundant methane oxidizing OTU belonging to *Methylocystis* was present throughout all sites corresponding to its prevalence in wetlands^71^. Its abundance correlated with pore water methane concentrations (Fig. S7) which corresponds to related studies^72,73^. It was proposed that historical contingencies (i.e., random events), rather than evolutionary acquired fitness underlie the variations in *Methylocystis* communities^74,75^. Based on this, we suggest that *Methylocystis* adapted to the changes associated to succession, including pH. The observation that the communities of methane oxidizers in this study are dominated by *Methylocystis*, with high abundances of *Methylomonas* and *Methyloferula* in the *Sphagnum*-sites, extends on related studies which showed that *Methylobacter* typically thrives in Arctic pH-neutral peatland soils^18,76^. Together with a number of studies focusing on the cultivation of methane oxidizers in *Sphagnum* peatlands^77–80^, this study further shows that the vegetation-associated methanotrophic community of Arctic peatlands is different from that of the peat soil (similar to what was discussed earlier for the overall microbiota) and that the low pH *Sphagnum* peatlands favor specific methanotrophic taxa such as *Methyloferula*.

### Distinct patterns of endophytic bacteria

We identified distinct patterns of putative endophytic bacteria for both *Sphagnum* and Amblystegiaceae. The different bacterial endophytes in Amblystegiaceae compared to *Sphagnum* likely reflect a direct influence of the moss taxa on the microbiota. It was suggested earlier that *Sphagnum* plants select for beneficial bacteria through secondary metabolites^32^. The cell wall of *Sphagnum* contains polysaccharides and lignin-like polymers^81^, and our data show that the cell wall composition of *Sphagnum* and Amblystegiaceae is basically the same (Table S1A). Further, pectin-like polymers represent a minor fraction of cell wall polysaccharides, providing the mosses with substantial cation exchange capacity (CEC)^81,82^ which is similar in both moss groups (Table S1A), corresponding to previous findings^13^. However, Amblystegiaceae have a greater proportion of lignin-like phenolic polymers compared to *Sphagnum*. Also, in *Sphagnum*-dominated sites the cation exchange maintains low pH. By contrast, the cation exchange does not reduce and control pH in Amblystegiaceae-dominated fens due to the substantial neutralization capacity of the mineral-rich groundwater. Besides a direct pH control, the cation exchange sites of *Sphagnum* pectin-like polymers inhibit microbial activity when these polymers hydrolyze and are released to the surroundings as so-called sphagnan^81^. Apart from the selection of beneficial microorganisms, *Sphagnum* protects itself against pathogens, e.g. via close association with antagonistic and antifungal bacteria or by release of antimicrobial substances^81–84^.

We further found that the putative endophytic microbiota was distinct from the epiphytic microbiota. It is known that bacterial endophytes can be host plant-specific promoting growth or health of their hosts^85,86^. Several OTUs, shown here to represent putative endophytes of *Sphagnum* or Amblystegiaceae, were previously reported as host plant-specific, e.g., Kineosporiaceae, Hyphomicrobiaceae, Intrasporangiaceae and Acidimicrobiales^87–90^. Endophytic microorganisms can be transferred from one generation to another, as shown for *Sphagnum*, or colonize the host from the surroundings after attraction by moss secreted chemical signals^31,40,91^. The inheritance and selection of potentially beneficial endophytes may provide an explanation for the distinct endophytic communities not only of *Sphagnum* but also of Amblystegiaceae.

### The core microbiota and its possible role for peatland succession

The total core microbiome of Amblystegiaceae and *Sphagnum* ecosystem samples was small compared to the total number of OTUs identified (49 vs. 13,799). However, the *Sphagnum* core microbiome in our study (142 OTUs) spanning Subarctic and temperate regions was similar in size to the alpine *Sphagnum* bog core microbiome (260 OTUs) reported elsewhere^30^. The sum of relative abundances of the OTUs in the total core microbiome was very high. Thus, this core community is a collection of few but highly abundant and thus, presumably, important members of the moss-associated microbial communities. On the other hand, the large numbers of low abundant OTUs that are not part of the core microbiomes identified in this study are possibly a result of local assemblies of microorganisms from the immediate surroundings. Notably, a major part of the total core microbiome were epiphytes on Amblystegiaceae, while dominant endophytes in *Sphagnum*. It needs to be emphasized that the patterns are dominated by the moss samples which reflects the sample design at least to some extent since we mainly collected mosses. Nevertheless, those patterns appear robust since the reference samples associate well with the moss samples (Fig. 4). Considering that these observed patterns of epi- and endophytes are consistent across large distances, many subsites, moss species and conditions, we hypothesize that *Sphagnum* recruited parts of the Amblystegiaceae microbiome during establishment in Amblystegiaceae peatlands, after which parts of the recruited microbiome adapted and became dominant endophytic members of the *Sphagnum* core microbiome that can be vertically transferred to the next generation as shown previously^31^. This way, over time, a *Sphagnum* core microbiome that originated in part from Amblystegiaceae, may have established. Alternatively, recruitment of microorganisms by *Sphagnum* is independent from peatland succession. If so, the presence of abundant OTUs that are dominant endophytes of *Sphagnum* and epiphytes of Amblystegiaceae is a coincidence and these bacteria dominate both systems because they are able to survive in both types of environments. However, considering that Amblystegiaceae and *Sphagnum* co-exist during peatland succession, a frequently occurring event through history^9,11,16^, it is possible that parts of the shared microbiome have transferred during times of co-existence. However, as the existence of an abundant core microbiome was first identified during the analysis of these data, the question of its role during succession is outside the scope of the current study.

## Conclusion

We have uncovered the identity of moss-associated microorganisms in both *Sphagnum* and Amblystegiaceae peatlands and show that the dominant part of the moss microbiota in northern peatlands is influenced by the plant host. Amblystegiaceae and *Sphagnum* mosses share a small but highly abundant bacterial core microbiome with members that are dominant endophytes in *Sphagnum* and epiphytes in Amblystegiaceae. Future work is needed to test whether this core microbiome is a result of transfers of epiphytic bacteria from Amblystegiaceae to *Sphagnum* during natural peatland succession from fens to bogs.

## Supplementary Information

Supplementary Information 1.

Supplementary Information 2.

Supplementary Information 3.

Supplementary Information 4.

Supplementary Information 5.

Supplementary Information 6.

Supplementary Information 7.

## Data Availability

Demultiplexed read sequence data has been deposited at NCBI/Genbank database under the BioProject PRJNA356121 with accession numbers SRR6442387- SRR6442509 for bacteria and SRR6442615-SRR6442637 for archaea.
