# Supplementary material for: Environmental patterns of brown moss- and Sphagnum-associated microbial communities
Source: Sci Rep. 2020 Dec 29;10:22412. doi: 10.1038/s41598-020-79773-2 (PMC7772339; doi:10.1038/s41598-020-79773-2)
Supplement: Supplementary file 7 — Supplementary Information 7. [file 41598_2020_79773_MOESM7_ESM.docx]

**Supplementary Information**

Environmental patterns of brown moss- and *Sphagnum*-associated microbial communities

Alexander Tøsdal Tveit^1*^, Andrea Kiss^2*^, Matthias Winkel^2^, Fabian Horn^2^, Tomáš Hájek^3^, Mette Marianne Svenning^1^, Dirk Wagner^2,4^, Susanne Liebner^2,5^

UiT The Arctic University of Norway, Department of Arctic and Marine Biology, Tromsø, Norway1, GFZ German Research Center for Geosciences, Section Geomicrobiology, Potsdam, Germany2, Faculty of Science, University of South Bohemia, České Budějovice, Czech Republic3, University of Potsdam, Institute of Geosciences, Potsdam, Germany^4^, University of Potsdam, Institute of Biochemistry and Biology, Potsdam, Germany^5^

*authors contributed equally to the study

**
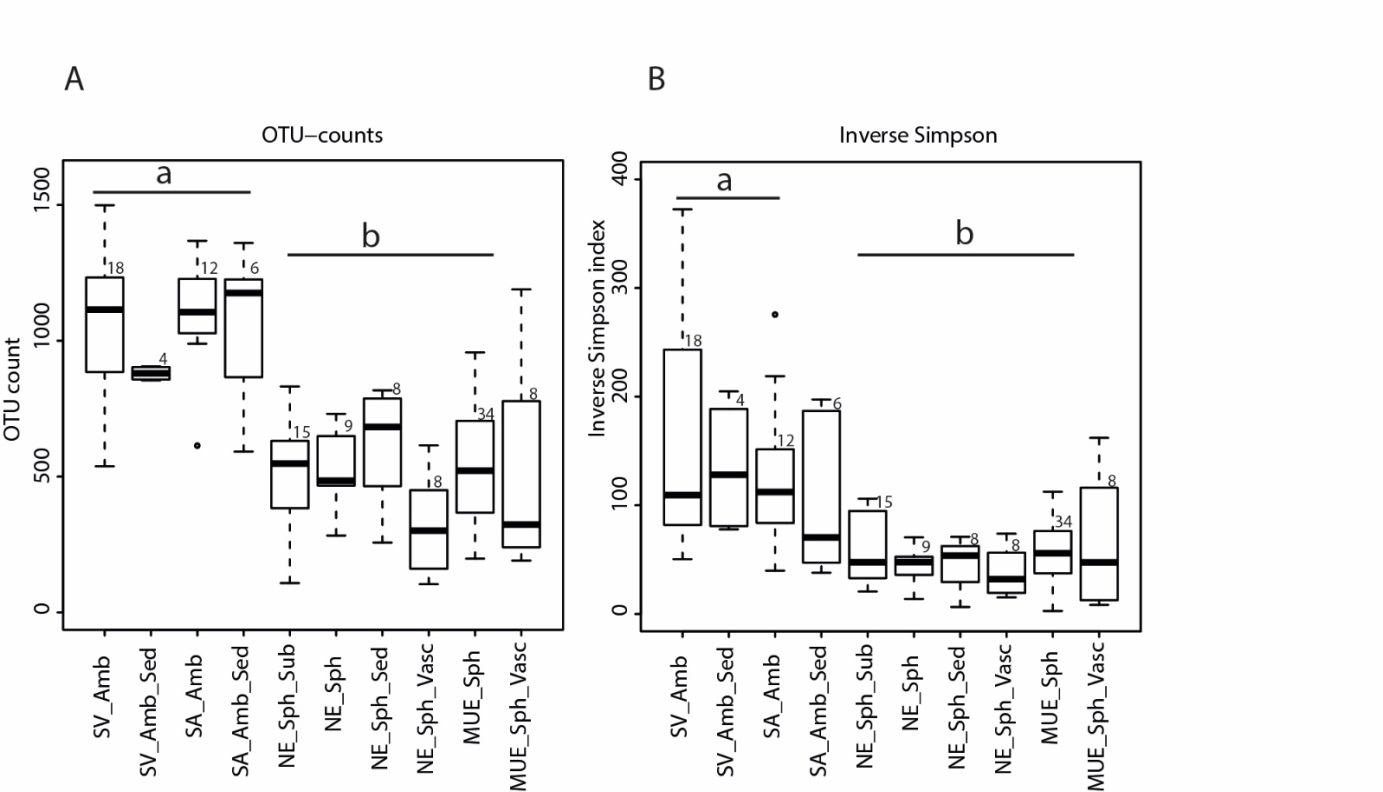
**

**Fig. S1:** **Box plots illustrating bacterial alpha-diversities for Amblystegiaceae (Amb), sediment references to Amblystegiaceae (Amb_Sed), *Sphagnum* (Sph), sediment references to *Sphagnum* (Sph_Sed) and vascular plant references to *Sphagnum* (Sph_Vasc).** A: Observed OTU; B: Calculated Inverse Simpson Index; a pairwise Mann-Whitney-Wilcoxon test (significance level set to 0.05) suggests that the mean diversities of all samples from group a are significantly different to those of group b. MUE_Sph_Vasc is not significantly different to any other group. Panel A: SA_AMb_Sed and NE_Sph_Sed not significantly different (p-val 0.02).


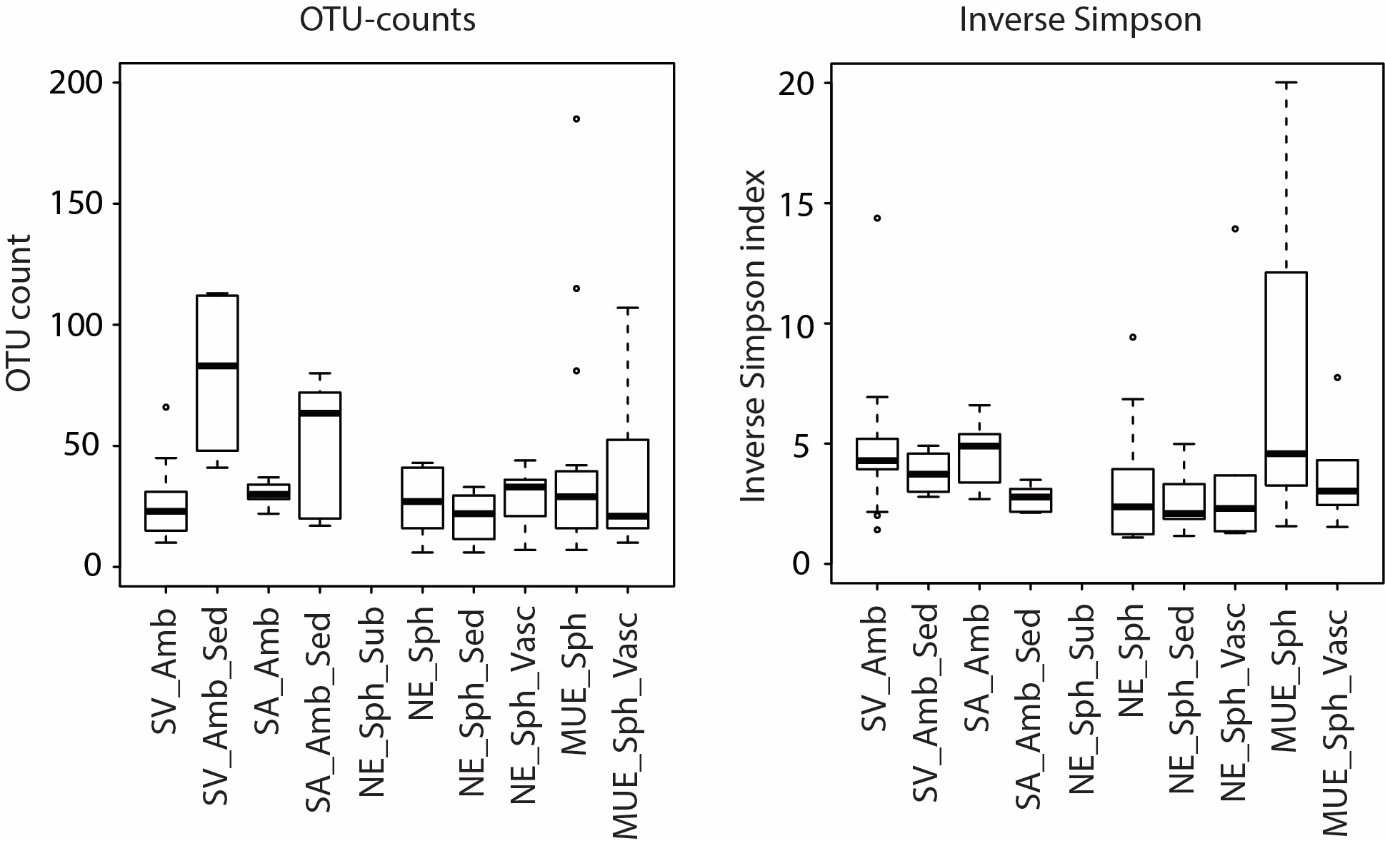


**Fig. S2:** **Box plots illustrating archaeal alpha-diversities for Amblystegiaceae (Amb), sediment references to Amblystegiaceae (Amb_Sed), *Sphagnum* (Sph), sediment references to *Sphagnum* (Sph_Sed) and vascular plant references to *Sphagnum* (Sph_Vasc).** A: Observed OTU; B: Calculated Inverse Simpson Index.

**
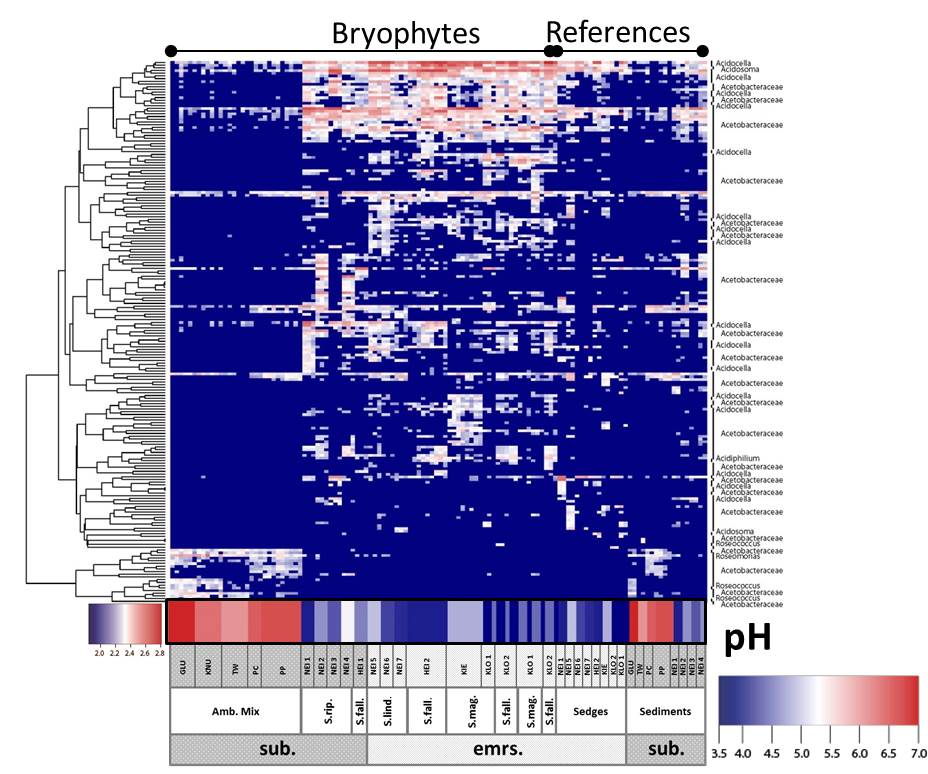
**

**Fig. S3:** **Heatmap overview of OTUs within Acetobacteraceae.** For each heatmap: The color intensity of the main heatmap (at the top left of both heatmaps) corresponds to the binary logarithm of the relative abundance of the OTU multiplied by 100,000. Pearson correlation was used as the basis for the hierarchical clustering of OTUs in the heatmap. The color intensity of the pH heatmap corresponds to the pH. The samples are sorted by ecosystem types and latitude from left to right. sub. = submerged. emrs. = emerged/above the water table. Amb. Mix. = a mix of *Amblystegiaceae*. S. rip. = *Sphagnum riparium*. S. fall. = *Sphagnum fallax.* S. mag = *Sphagnum magellanicum*. S. lind. = *Sphagnum lindbergii.*


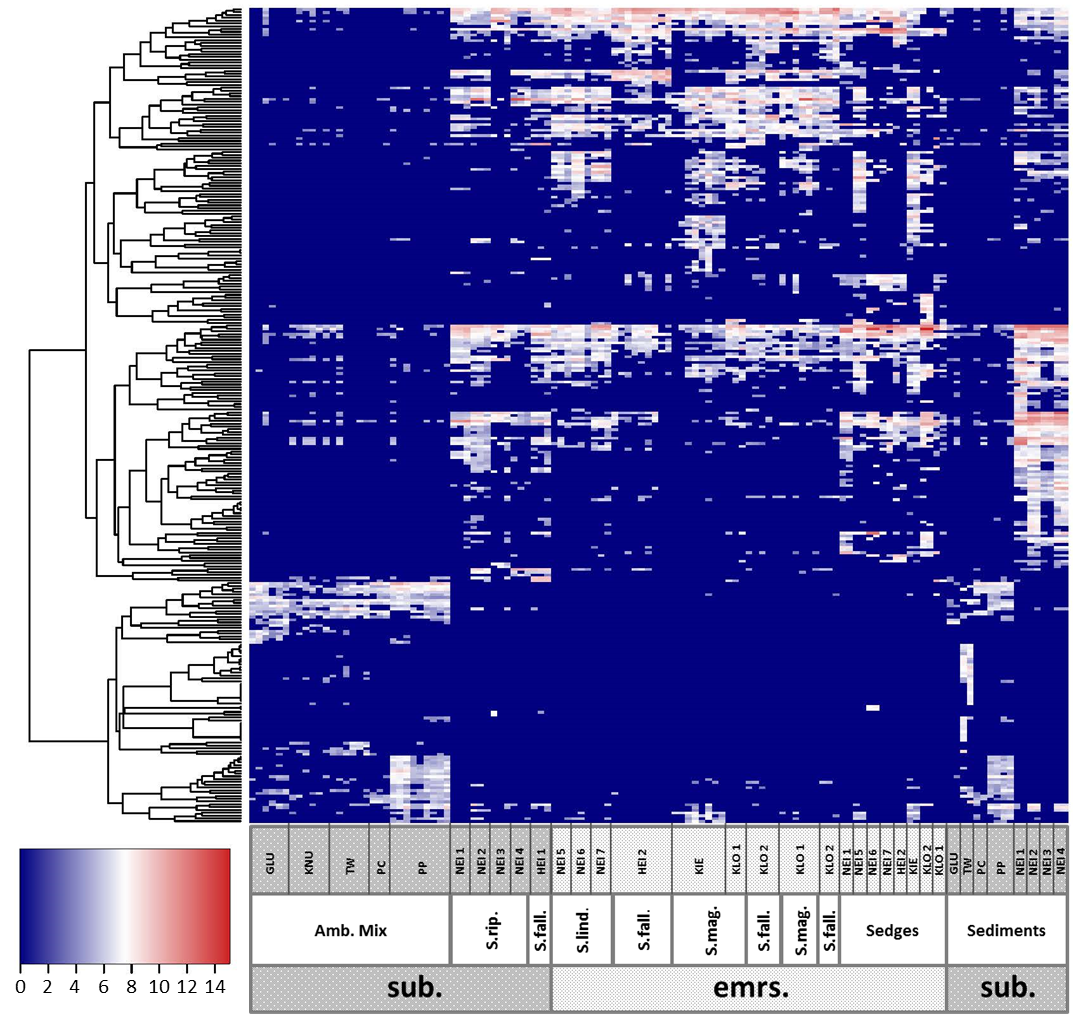


**Fig. S4**: **Heatmap overview of OTUs within Acidobacteria.** The color intensity of the main heatmap corresponds to the binary logarithm of the average relative abundance of the OTU multiplied by 100,000. Pearson correlation was used as the basis for the hierarchical clustering of OTUs in the heatmap. The samples are sorted by ecosystem types and latitude from left to right. sub. = submerged. emrs. = emerged/above the water table. Amb. Mix. = a mix of *Amblystegiaceae*. S. rip. = *Sphagnum riparium*. S. fall. = *Sphagnum fallax.* S. mag = *Sphagnum magellanicum*. S. lind. = *Sphagnum lindbergii.*


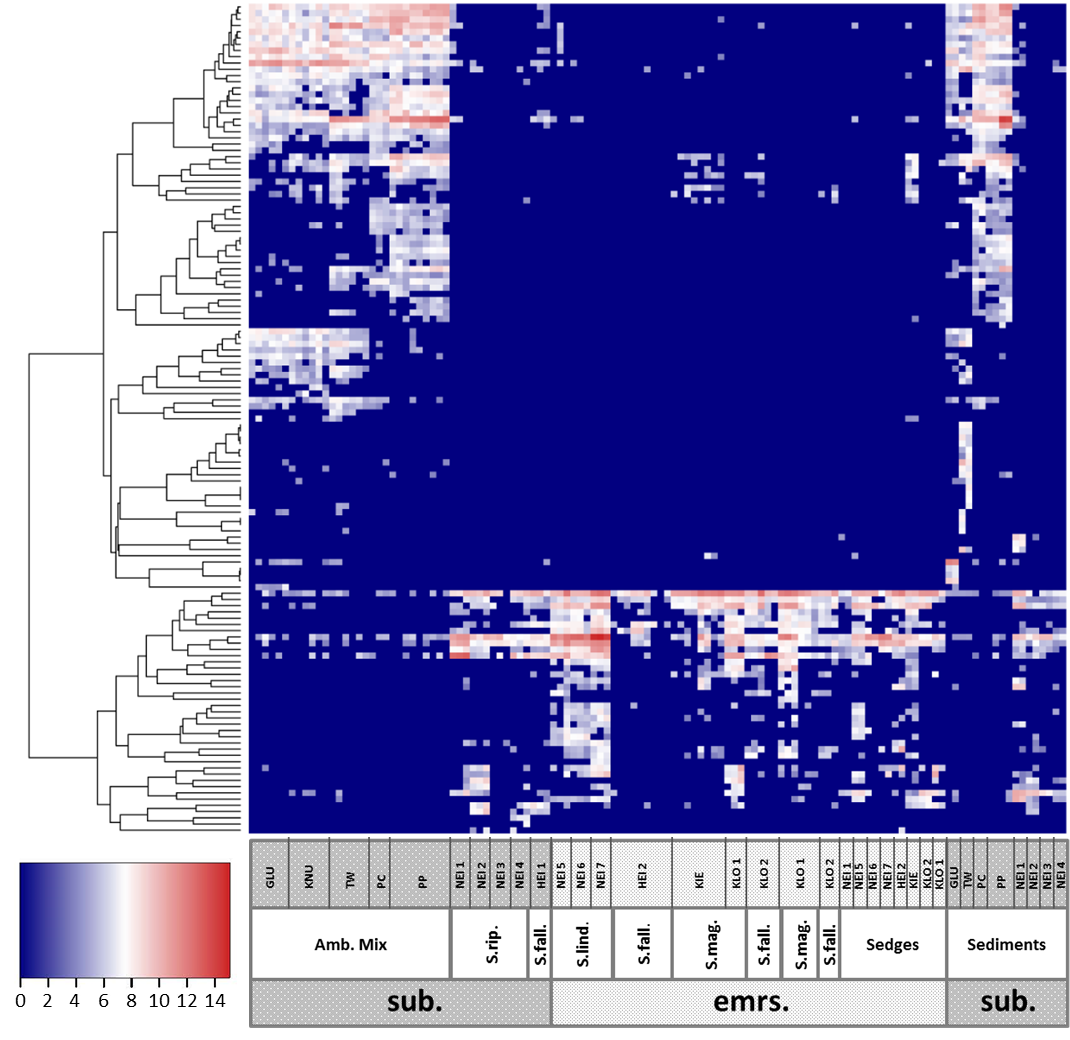


**Fig. S5:** **Heatmap overview of OTUs within Acidimicrobiales.** The color intensity of the main heatmap corresponds to the binary logarithm of the average relative abundance of the OTU multiplied by 100,000. Pearson correlation was used as the basis for the hierarchical clustering of OTUs in the heat map. The samples are sorted by ecosystem types and latitude from left to right. sub. = submerged. emrs. = emerged/above the water table. Amb. Mix. = a mix of *Amblystegiaceae*. S. rip. = *Sphagnum riparium*. S. fall. = *Sphagnum fallax.* S. mag = *Sphagnum magellanicum*. S. lind. = *Sphagnum lindbergii*.


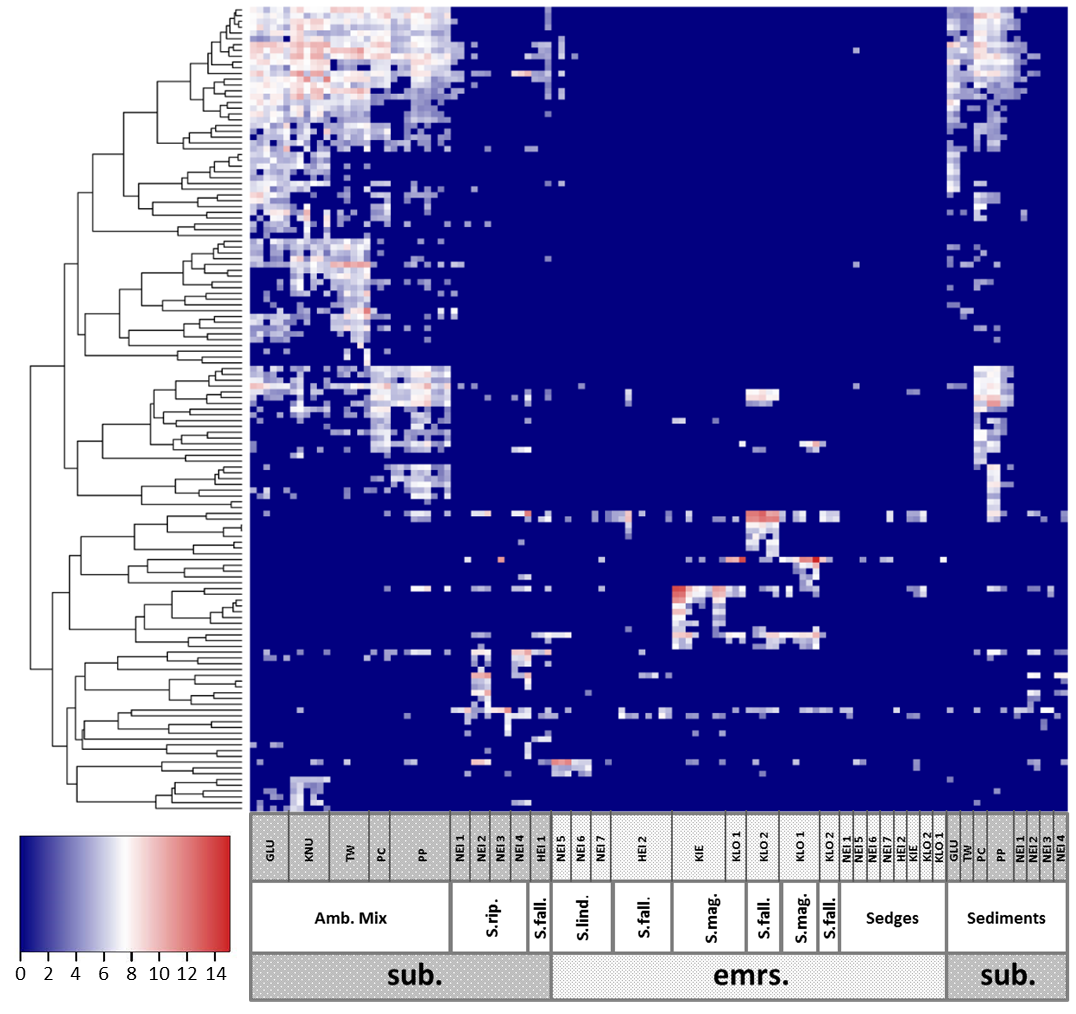


**Fig. S6**: **Heatmap overview of OTUs within Cyanobacteria.** The color intensity of the main heatmap corresponds to the binary logarithm of the average relative abundance of the OTU multiplied by 100,000. Pearson correlation was used as the basis for the hierarchical clustering of OTUs in the heatmap. The samples are sorted by ecosystem types and latitude from left to right. sub. = submerged. emrs. = emerged/above the water table. Amb. Mix. = a mix of *Amblystegiaceae*. S. rip. = *Sphagnum riparium*. S. fall. = *Sphagnum fallax.* S. mag = *Sphagnum magellanicum*. S. lind. = *Sphagnum lindbergii*.

**
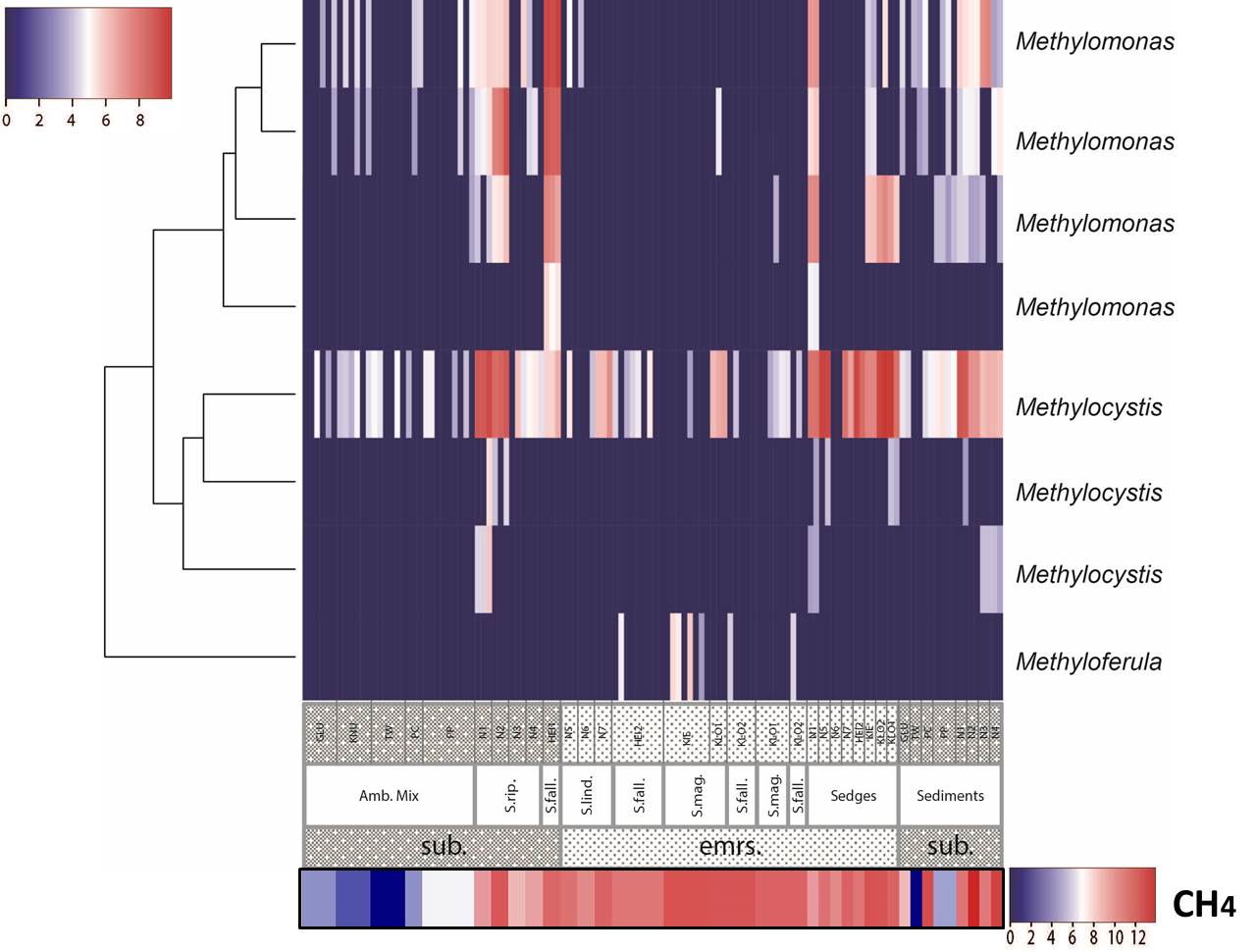
**

**Fig. S7:** **Heat map overview of OTUs within methanotrophic bacteria.** The color intensity of the main heatmap (at the top left of both heatmaps) corresponds to the binary logarithm of the relative abundance of the OTU multiplied by 100,000. Pearson correlation was used as the basis for the hierarchical clustering of OTUs in the heatmap. The color intensity of the CH_4_ heatmap corresponds to the binary logarithm of the pore water CH_4_ concentration. The samples are sorted by ecosystem types and latitude from left to right. sub. = submerged. emrs. = emerged/above the water table. Amb. Mix. = a mix of *Amblystegiaceae*. S. rip. = *Sphagnum riparium*. S. fall. = *Sphagnum fallax.* S. mag = *Sphagnum magellanicum*. S. Lind. = *Sphagnum lindbergii.* All OTUs taxonomically assigned as potential MOB (it means all Methylocystaceae and all Methylococcales) were manually blasted in NCBI for their closest relative. Only OTUs that were confirmed to be MOB were used for constructing this heatmap.


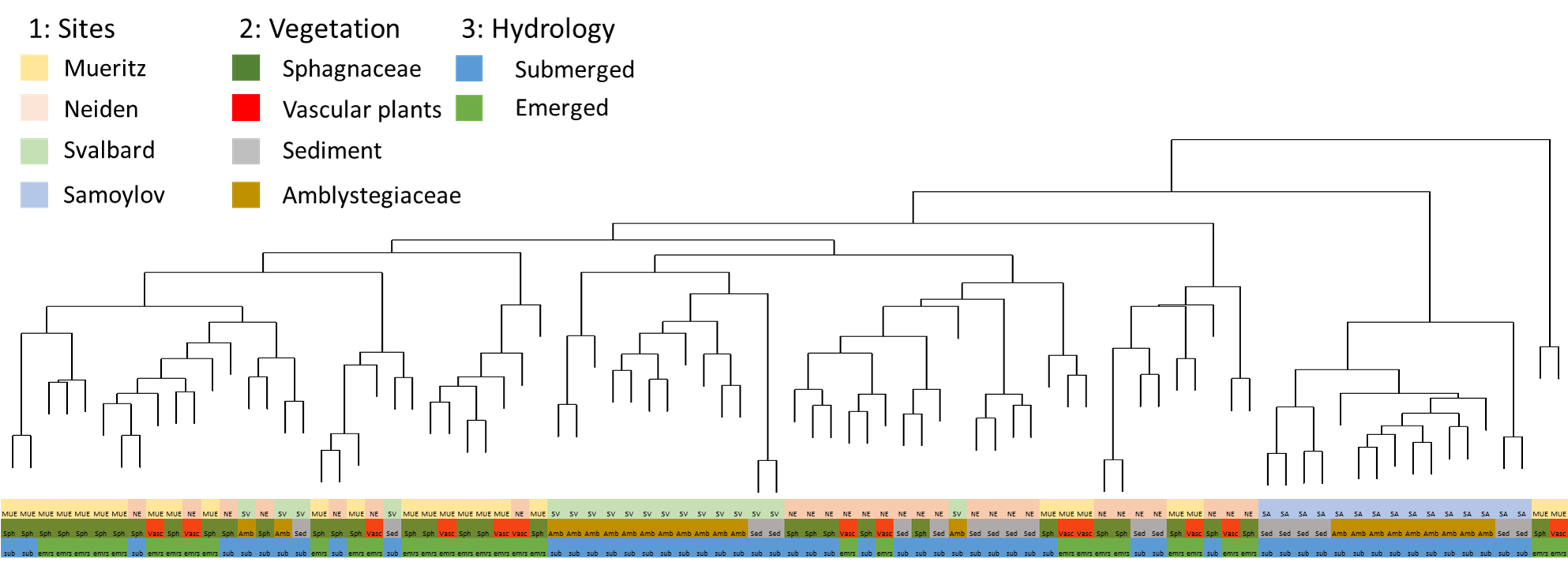


**Fig. S8**: Dendrogram showing the clustering of archaeal communities (OTU at 97% sequence similarity) in relation to the characteristics of their respective environments. Each node of the dendrogram corresponds to the community profile of a moss, vascular plant or sediment sample. All possible pairwise pearson correlation factors were calculated from the community profiles and the resulting distance matrix used to cluster the samples applying the agnes hierarchical clustering algorithm.


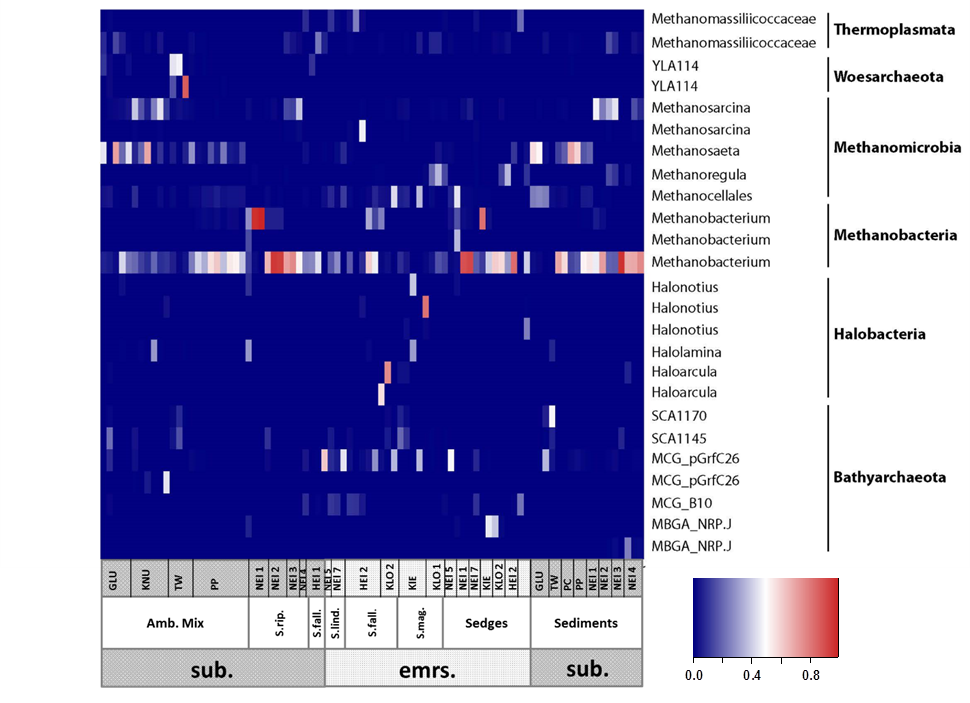


**Fig. S9**: **Heatmap overview of the OTUs within archaea at above 0.1% relative abundance (out of total sequences assigned to Archaea) in one or more samples.** The color intensity corresponds to the binary logarithm of the average relative abundance of the OTU multiplied by 100,000. Pearson correlation was used as the basis for the hierarchical clustering of OTUs in the heatmap. The samples are sorted by ecosystem types and latitude from left to right. sub. = submerged. emrs. = emerged/above the water table. Amb. Mix. = a mix of *Amblystegiaceae*. S. rip. = *Sphagnum riparium*. S. fall. = *Sphagnum fallax.* S. mag = *Sphagnum magellanicum*. S. lind. = *Sphagnum lindbergii*.

**Supplementary Table S1A: Environmental variables and meta-data of all samples.** KL=Klason lignin which is the fraction of lignin that is insoluble in acids (given in % of dry mass); sol-KL=acid-soluble Klason lignin (given in % of dry mass); HC=content of hemicellulose (given in % of dry mass); CEC= cation exchange capacity (given in µeq/g); oxygen in mg L^-1^; CH_4_ concentration in µM; DOC in mg L^-1^; C and N in % dry weight; water content in %.

| Sample ID | Name | StudySite | SubSite | Taxon | Taxon-2 | Hydrol | Type | System | pH | CH4 | DOC | Oxygen | Temp | CEC | KL | sol-KL | HC | N | C | C:N | Water cont |
| --- | --- | --- | --- | --- | --- | --- | --- | --- | --- | --- | --- | --- | --- | --- | --- | --- | --- | --- | --- | --- | --- |
| 1 | GLU1 endo | SV | GLU 1 | Amb | Amb | sub | endo | amb | 7,00 | 7,04 | 4,40 | 8,57 | 8,93 | 668,20 | 35,10 | 2,00 | 32,90 | 1,09 | 30,75 | 27,92 | 51,10 |
| 2 | GLU1 endo | SV | GLU 1 | Amb | Amb | sub | endo | amb | 7,00 | 7,04 | 4,40 | 8,57 | 8,93 | 668,20 | 35,10 | 2,00 | 32,90 | 1,09 | 30,75 | 27,92 | 51,10 |
| 3 | TW2 endo | SV | TW 2 | Amb | Amb | sub | endo | amb | 5,90 | 0,84 | 1,08 | 6,62 | 11,00 | 430,50 | 48,00 | 1,40 | 56,40 | 1,27 | 28,22 | 22,20 | 51,70 |
| 4 | TW2 endo | SV | TW 2 | Amb | Amb | sub | endo | amb | 5,90 | 0,84 | 1,08 | 6,62 | 11,00 | 430,50 | 48,00 | 1,40 | 56,40 | 1,27 | 28,22 | 22,20 | 51,70 |
| 5 | NEI1 endo | NE | 1 | Sph | S.rip | sub | endo | sph | 3,75 | 151,60 | 59,98 | 3,85 | 12,16 | 461,90 | 4,80 | 5,10 | 46,00 | 0,76 | 45,88 | 60,11 | 78,80 |
| 6 | NEI1 endo | NE | 1 | Sph | S.rip | sub | endo | sph | 3,75 | 151,60 | 59,98 | 3,85 | 12,16 | 461,90 | 4,80 | 5,10 | 46,00 | 0,76 | 45,88 | 60,11 | 78,80 |
| 7 | NEI3 endo | NE | 6 | Sph | S.rip | sub | endo | sph | 4,03 | 95,06 | 31,45 | 7,23 | 9,59 | 468,90 | 10,30 | 4,20 | 48,30 | 1,29 | 44,46 | 34,38 | 93,80 |
| 8 | NEI3 endo | NE | 6 | Sph | S.rip | sub | endo | sph | 4,03 | 95,06 | 31,45 | 7,23 | 9,59 | 468,90 | 10,30 | 4,20 | 48,30 | 1,29 | 44,46 | 34,38 | 93,80 |
| 9 | GLU2 endo | SV | GLU 2 | Amb | Amb | sub | endo | amb | 7,00 | 7,04 | 4,40 | 8,57 | 8,93 | 668,20 | 35,10 | 2,00 | 32,90 | 1,09 | 30,75 | 27,92 | 51,10 |
| 10 | GLU2 endo | SV | GLU 2 | Amb | Amb | sub | endo | amb | 7,00 | 7,04 | 4,40 | 8,57 | 8,93 | 668,20 | 35,10 | 2,00 | 32,90 | 1,09 | 30,75 | 27,92 | 51,10 |
| 11 | NEI6 endo | NE | 2-2 | Sph | S.lind | emrs | endo | sph | 3,92 | 198,32 | NA | NA | 13,82 | 729,40 | 7,60 | 7,60 | 49,10 | 0,47 | 45,03 | 95,26 | 79,10 |
| 12 | NEI6 endo | NE | 2-2 | Sph | S.lind | emrs | endo | sph | 3,92 | 198,32 | NA | NA | 13,82 | 729,40 | 7,60 | 7,60 | 49,10 | 0,47 | 45,03 | 95,26 | 79,10 |
| 13 | NEI4 endo | NE | 4-2 | Sph | S.rip | sub | endo | sph | 4,95 | 136,30 | 18,10 | 2,24 | 12,58 | 386,30 | 14,70 | 3,60 | 51,30 | 2,30 | 41,80 | 18,16 | 93,60 |
| 14 | NEI4 endo | NE | 4-2 | Sph | S.rip | sub | endo | sph | 4,95 | 136,30 | 18,10 | 2,24 | 12,58 | 386,30 | 14,70 | 3,60 | 51,30 | 2,30 | 41,80 | 18,16 | 93,60 |
| 15 | NEI2 endo | NE | 4 | Sph | S.rip | sub | endo | sph | 4,35 | 454,62 | NA | NA | 13,73 | 359,10 | NA | NA | NA | 1,76 | 43,28 | 24,61 | 93,70 |
| 16 | NEI2 endo | NE | 4 | Sph | S.rip | sub | endo | sph | 4,35 | 454,62 | NA | NA | 13,73 | 359,10 | NA | NA | NA | 1,76 | 43,28 | 24,61 | 93,70 |
| 17 | NEI5 endo | NE | 2 | Sph | S.lind | emrs | endo | sph | 4,63 | 296,92 | 83,98 | 4,32 | 12,45 | 580,90 | 16,40 | 6,80 | 52,40 | 0,49 | 45,90 | 93,78 | 79,10 |
| 18 | NEI5 endo | NE | 2 | Sph | S.lind | emrs | endo | sph | 4,63 | 296,92 | 83,98 | 4,32 | 12,45 | 580,90 | 16,40 | 6,80 | 52,40 | 0,49 | 45,90 | 93,78 | 79,10 |
| 19 | KLO2 endo | MUE | KLO-m 5 | Sph | S.fall | emrs | endo | sph | 4,15 | 359,64 | 45,30 | 1,64 | 16,50 | 763,20 | 14,70 | 4,90 | 51,20 | 0,83 | 45,47 | 54,74 | 92,00 |
| 20 | KLO2 endo | MUE | KLO-m 5 | Sph | S.fall | emrs | endo | sph | 4,15 | 359,64 | 45,30 | 1,64 | 16,50 | 763,20 | 14,70 | 4,90 | 51,20 | 0,83 | 45,47 | 54,74 | 92,00 |
| 21 | KLO1 endo | MUE | KLO-o 3 | Sph | S.mag | emrs | endo | sph | 3,60 | 461,79 | 42,15 | 1,64 | 16,16 | 677,30 | 19,50 | 5,30 | 58,00 | 1,02 | 46,93 | 47,30 | 90,80 |
| 22 | KLO1 endo | MUE | KLO-o 3 | Sph | S.mag | emrs | endo | sph | 3,60 | 461,79 | 42,15 | 1,64 | 16,16 | 677,30 | 19,50 | 5,30 | 58,00 | 1,02 | 46,93 | 47,30 | 90,80 |
| 23 | HEI2 endo | MUE | HEI 2 | Sph | S.fall | emrs | endo | sph | 3,75 | 277,34 | 227,50 | NA | 15,84 | 574,30 | 18,90 | 4,50 | 59,70 | 1,30 | 45,60 | 35,00 | 92,20 |
| 24 | HEI2 endo | MUE | HEI 2 | Sph | S.fall | emrs | endo | sph | 3,75 | 277,34 | 227,50 | NA | 15,84 | 574,30 | 18,90 | 4,50 | 59,70 | 1,30 | 45,60 | 35,00 | 92,20 |
| 25 | KIE1 endo | MUE | KIE 1 | Sph | S.mag | emrs | endo | sph | 4,53 | 482,09 | NA | NA | NA | 783,10 | 28,30 | 4,20 | 58,50 | 0,82 | 45,79 | 56,64 | 92,00 |
| 26 | KIE1 endo | MUE | KIE 1 | Sph | S.mag | emrs | endo | sph | 4,53 | 482,09 | NA | NA | NA | 783,10 | 28,30 | 4,20 | 58,50 | 0,82 | 45,79 | 56,64 | 92,00 |
| 27 | KLO2 endo | MUE | KLO-m 4 | Sph | S.fall | emrs | endo | sph | 3,60 | 461,79 | 42,15 | 1,64 | 16,16 | 677,30 | 19,50 | 5,30 | 58,00 | 1,02 | 46,93 | 47,30 | 90,80 |
| 28 | KLO2 endo | MUE | KLO-m 4 | Sph | S.fall | emrs | endo | sph | 3,60 | 461,79 | 42,15 | 1,64 | 16,16 | 677,30 | 19,50 | 5,30 | 58,00 | 1,02 | 46,93 | 47,30 | 90,80 |
| 31 | NEI7 endo | NE | 2-3 | Sph | S.lind | emrs | endo | sph | 3,80 | 365,89 | NA | NA | 11,48 | 683,30 | 6,20 | 6,50 | 60,00 | 1,00 | 85,95 | 85,87 | 79,10 |
| 32 | NEI7 endo | NE | 2-3 | Sph | S.lind | emrs | endo | sph | 3,80 | 365,89 | NA | NA | 11,48 | 683,30 | 6,20 | 6,50 | 60,00 | 1,00 | 85,95 | 85,87 | 79,10 |
| 33 | KLO1 endo | MUE | KLO-o 1 | Sph | S.mag | emrs | endo | sph | 4,15 | 359,64 | 45,30 | 1,64 | 16,50 | 763,20 | 14,70 | 4,90 | 51,20 | 0,83 | 45,47 | 54,74 | 92,00 |
| 34 | KLO1 endo | MUE | KLO-o 1 | Sph | S.mag | emrs | endo | sph | 4,15 | 359,64 | 45,30 | 1,64 | 16,50 | 763,20 | 14,70 | 4,90 | 51,20 | 0,83 | 45,47 | 54,74 | 92,00 |
| 35 | KLO1 endo | MUE | KLO-o 2 | Sph | S.mag | emrs | endo | sph | 4,15 | 359,64 | 45,30 | 1,64 | 16,50 | 763,20 | 14,70 | 4,90 | 51,20 | 0,83 | 45,47 | 54,74 | 92,00 |
| 36 | KLO1 endo | MUE | KLO-o 2 | Sph | S.mag | emrs | endo | sph | 4,15 | 359,64 | 45,30 | 1,64 | 16,50 | 763,20 | 14,70 | 4,90 | 51,20 | 0,83 | 45,47 | 54,74 | 92,00 |
| 37 | KLO2 endo | MUE | KLO-m 6 | Sph | S.fall | emrs | endo | sph | 3,60 | 461,79 | 42,15 | 1,64 | 16,16 | 677,30 | 19,50 | 5,30 | 58,00 | 1,02 | 46,93 | 47,30 | 90,80 |
| 38 | KLO2 endo | MUE | KLO-m 6 | Sph | S.fall | emrs | endo | sph | 3,60 | 461,79 | 42,15 | 1,64 | 16,16 | 677,30 | 19,50 | 5,30 | 58,00 | 1,02 | 46,93 | 47,30 | 90,80 |
| 41 | KIE2 endo | MUE | KIE 2 | Sph | S.mag | emrs | endo | sph | 4,53 | 482,09 | NA | NA | NA | 783,10 | 28,30 | 4,20 | 58,50 | 0,82 | 45,79 | 56,64 | 92,00 |
| 45 | TW1 endo | SV | TW 1 | Amb | Amb | sub | endo | amb | 5,90 | 0,84 | 1,08 | 6,62 | 11,00 | 430,50 | 48,00 | 1,40 | 56,40 | 1,27 | 28,22 | 22,20 | 51,70 |
| 46 | TW1 endo | SV | TW 1 | Amb | Amb | sub | endo | amb | 5,90 | 0,84 | 1,08 | 6,62 | 11,00 | 430,50 | 48,00 | 1,40 | 56,40 | 1,27 | 28,22 | 22,20 | 51,70 |
| 47 | KIE3 endo | MUE | KIE 3 | Sph | S.mag | emrs | endo | sph | 4,53 | 482,09 | NA | NA | NA | 783,10 | 28,30 | 4,20 | 58,50 | 0,82 | 45,79 | 56,64 | 92,00 |
| 48 | KIE3 endo | MUE | KIE 3 | Sph | S.mag | emrs | endo | sph | 4,53 | 482,09 | NA | NA | NA | 783,10 | 28,30 | 4,20 | 58,50 | 0,82 | 45,79 | 56,64 | 92,00 |
| 49 | PP1 endo | SA | SuScor a | Amb | Scor | sub | endo | amb | 6,58 | 27,47 | 4,00 | 5,45 | 12,65 | 610,00 | 14,00 | 1,60 | 42,20 | 0,85 | 33,99 | 39,93 | 57,10 |
| 50 | PP1 endo | SA | SuScor a | Amb | Scor | sub | endo | amb | 6,58 | 27,47 | 4,00 | 5,45 | 12,65 | 610,00 | 14,00 | 1,60 | 42,20 | 0,85 | 33,99 | 39,93 | 57,10 |
| 51 | PP1 endo | SA | SuScor b | Amb | Scor | sub | endo | amb | 6,58 | 27,47 | 4,00 | 5,45 | 12,65 | 610,00 | 14,00 | 1,60 | 42,20 | 0,85 | 33,99 | 39,93 | 57,10 |
| 52 | PP1 endo | SA | SuScor b | Amb | Scor | sub | endo | amb | 6,58 | 27,47 | 4,00 | 5,45 | 12,65 | 610,00 | 14,00 | 1,60 | 42,20 | 0,85 | 33,99 | 39,93 | 57,10 |
| 53 | PC endo | SA | S9 | Amb | Amb | sub | endo | amb | 6,45 | 7,03 | NA | NA | NA | 566,00 | 27,50 | 1,70 | 37,30 | 1,22 | 64,12 | 52,76 | 57,10 |
| 54 | PC endo | SA | S9 | Amb | Amb | sub | endo | amb | 6,45 | 7,03 | NA | NA | NA | 566,00 | 27,50 | 1,70 | 37,30 | 1,22 | 64,12 | 52,76 | 57,10 |
| 55 | HEI2 endo | MUE | HEI 1 | Sph | S.fall | emrs | endo | sph | 3,75 | 277,34 | 227,50 | NA | 15,84 | 574,30 | 18,90 | 4,50 | 59,70 | 1,30 | 45,60 | 35,00 | 92,20 |
| 56 | HEI2 endo | MUE | HEI 1 | Sph | S.fall | emrs | endo | sph | 3,75 | 277,34 | 227,50 | NA | 15,84 | 574,30 | 18,90 | 4,50 | 59,70 | 1,30 | 45,60 | 35,00 | 92,20 |
| 57 | HEI2 endo | MUE | HEI 3 | Sph | S.fall | emrs | endo | sph | 3,75 | 277,34 | 227,50 | NA | 15,84 | 574,30 | 18,90 | 4,50 | 59,70 | 1,30 | 45,60 | 35,00 | 92,20 |
| 58 | HEI2 endo | MUE | HEI 3 | Sph | S.fall | emrs | endo | sph | 3,75 | 277,34 | 227,50 | NA | 15,84 | 574,30 | 18,90 | 4,50 | 59,70 | 1,30 | 45,60 | 35,00 | 92,20 |
| 59 | HEI1 endo | MUE | HEI 4 | Sph | S.fall | sub | endo | sph | 4,26 | 354,18 | 72,90 | 2,85 | 14,93 | 401,00 | NA | NA | NA | 1,50 | 48,90 | 32,00 | 97,20 |
| 60 | HEI1 endo | MUE | HEI 4 | Sph | S.fall | sub | endo | sph | 4,26 | 354,18 | 72,90 | 2,85 | 14,93 | 401,00 | NA | NA | NA | 1,50 | 48,90 | 32,00 | 97,20 |
| 67 | KLO2 Vasc | MUE | KLO-m 6 | Vasc | Cx | emrs | Ref | sph | 3,60 | 461,79 | 42,15 | 1,64 | 16,16 | NA | 39,30 | 2,50 | 58,90 | 1,34 | 50,60 | 37,90 | NA |
| 68 | KLO2 Vasc | MUE | KLO-m 6 | Vasc | Cx | emrs | Ref | sph | 3,60 | 461,79 | 42,15 | 1,64 | 16,16 | NA | 39,30 | 2,50 | 58,90 | 1,34 | 50,60 | 37,90 | NA |
| 69 | NEI7 Vasc | NE | 2-3 | Vasc | Vasc | emrs | Ref | sph | 3,80 | 365,89 | NA | NA | 11,48 | NA | 26,40 | 1,40 | 46,90 | 1,08 | 47,13 | 43,80 | NA |
| 70 | NEI7 Vasc | NE | 2-3 | Vasc | Vasc | emrs | Ref | sph | 3,80 | 365,89 | NA | NA | 11,48 | NA | 26,40 | 1,40 | 46,90 | 1,08 | 47,13 | 43,80 | NA |
| 71 | KIE Vasc | MUE | KIE 1 | Vasc | Cx | emrs | Ref | sph | 4,53 | 482,09 | NA | NA | NA | NA | 45,10 | 1,80 | 58,70 | 0,78 | 49,90 | 64,54 | NA |
| 72 | KIE Vasc | MUE | KIE 1 | Vasc | Cx | emrs | Ref | sph | 4,53 | 482,09 | NA | NA | NA | NA | 45,10 | 1,80 | 58,70 | 0,78 | 49,90 | 64,54 | NA |
| 73 | NEI6 Vasc | NE | 2-2 | Vasc | Cx | emrs | Ref | sph | 3,92 | 198,32 | NA | NA | 13,82 | NA | 37,60 | 1,60 | 61,10 | 0,57 | 47,70 | 83,75 | NA |
| 74 | NEI6 Vasc | NE | 2-2 | Vasc | Cx | emrs | Ref | sph | 3,92 | 198,32 | NA | NA | 13,82 | NA | 37,60 | 1,60 | 61,10 | 0,57 | 47,70 | 83,75 | NA |
| 75 | PP2 endo | SA | SuCar | Amb | Scor | sub | endo | amb | 6,58 | 27,47 | 4,00 | 5,45 | 12,65 | 569,00 | 48,20 | 1,30 | 40,50 | 0,93 | 31,46 | 33,73 | 57,10 |
| 76 | PP2 endo | SA | SuCar | Amb | Scor | sub | endo | amb | 6,58 | 27,47 | 4,00 | 5,45 | 12,65 | 569,00 | 48,20 | 1,30 | 40,50 | 0,93 | 31,46 | 33,73 | 57,10 |
| 77 | HEI2 Vasc | MUE | HEI 1 | Vasc | Erio | emrs | Ref | sph | 3,75 | 277,34 | 227,50 | NA | 15,84 | NA | 43,10 | 2,00 | 53,10 | 1,50 | 48,80 | 33,50 | NA |
| 78 | HEI2 Vasc | MUE | HEI 1 | Vasc | Erio | emrs | Ref | sph | 3,75 | 277,34 | 227,50 | NA | 15,84 | NA | 43,10 | 2,00 | 53,10 | 1,50 | 48,80 | 33,50 | NA |
| 86 | PP2 epi | SA | SuCar | Amb | Scor | sub | epi | amb | 6,58 | 27,47 | 4,00 | 5,45 | 12,65 | 569,00 | 48,20 | 1,30 | 40,50 | 0,93 | 31,46 | 33,73 | 57,10 |
| 89 | KLO1 epi | MUE | KLO-o 1 | Sph | S.mag | emrs | epi | sph | 4,15 | 359,64 | 45,30 | 1,64 | 16,50 | 763,20 | 14,70 | 4,90 | 51,20 | 0,83 | 45,47 | 54,74 | 92,00 |
| 90 | KLO1 epi | MUE | KLO-o 2 | Sph | S.mag | emrs | epi | sph | 4,15 | 359,64 | 45,30 | 1,64 | 16,50 | 763,20 | 14,70 | 4,90 | 51,20 | 0,83 | 45,47 | 54,74 | 92,00 |
| 92 | KLO2 epi | MUE | KLO-m 3 | Sph | S.fall | emrs | epi | sph | 3,60 | 461,79 | 42,15 | 1,64 | 16,16 | 677,30 | 19,50 | 5,30 | 58,00 | 1,02 | 46,93 | 47,30 | 90,80 |
| 93 | KLO2 epi | MUE | KLO-m 4 | Sph | S.fall | emrs | epi | sph | 3,60 | 461,79 | 42,15 | 1,64 | 16,16 | 677,30 | 19,50 | 5,30 | 58,00 | 1,02 | 46,93 | 47,30 | 90,80 |
| 94 | KLO2 epi | MUE | KLO-m 5 | Sph | S.fall | emrs | epi | sph | 4,15 | 359,64 | 45,30 | 1,64 | 16,50 | 763,20 | 14,70 | 4,90 | 51,20 | 0,83 | 45,47 | 54,74 | 92,00 |
| 98 | NEI1 epi | NE | 1 | Sph | S.rip | sub | epi | sph | 3,75 | 151,60 | 59,98 | 3,85 | 12,16 | 461,90 | 4,80 | 5,10 | 46,00 | 0,76 | 45,88 | 60,11 | 78,80 |
| 99 | NEI5 epi | NE | 2 | Sph | S.lind | emrs | epi | sph | 4,63 | 296,92 | 83,98 | 4,32 | 12,45 | 580,90 | 16,40 | 6,80 | 52,40 | 0,49 | 45,90 | 93,78 | 79,10 |
| 101 | NEI6 epi | NE | 2-2 | Sph | S.lind | emrs | epi | sph | 3,92 | 198,32 | NA | NA | 13,82 | 729,40 | 7,60 | 7,60 | 49,10 | 0,47 | 45,03 | 95,26 | 79,10 |
| 102 | NEI7 epi | NE | 2-3 | Sph | S.rip | emrs | epi | sph | 3,80 | 365,89 | NA | NA | 11,48 | 683,30 | 6,20 | 6,50 | 60,00 | 1,00 | 85,95 | 85,87 |  |
| 103 | NEI2 epi | NE | 4 | Sph | S.rip | sub | epi | sph | 4,35 | 454,62 | NA | NA | 13,73 | 359,10 | NA | NA | NA | 1,76 | 43,28 | 24,61 | 93,70 |
| 105 | NEI4 epi | NE | 4-2 | Sph | S.rip | sub | epi | sph | 4,95 | 136,30 | 18,10 | 2,24 | 12,58 | 386,30 | 14,70 | 3,60 | 51,30 | 2,30 | 41,80 | 18,16 | 93,60 |
| 106 | NEI3 epi | NE | 6 | Sph | S.rip | sub | epi | sph | 4,03 | 95,06 | 31,45 | 7,23 | 9,59 | 468,90 | 10,30 | 4,20 | 48,30 | 1,29 | 44,46 | 34,38 | 93,80 |
| 107 | GLU1 epi | SV | GLU 1 | Amb | Amb | sub | epi | amb | 7,00 | 7,04 | 4,40 | 8,57 | 8,93 | 668,20 | 35,10 | 2,00 | 32,90 | 1,09 | 30,75 | 27,92 | 51,10 |
| 109 | GLU2 epi | SV | GLU 2 | Amb | Amb | sub | epi | amb | 7,00 | 7,04 | 4,40 | 8,57 | 8,93 | 668,20 | 35,10 | 2,00 | 32,90 | 1,09 | 30,75 | 27,92 | 51,10 |
| 111 | HEI2 epi | MUE | HEI 1 | Sph | S.fall | emrs | epi | sph | 3,75 | 277,34 | 227,50 | NA | 15,84 | 574,30 | 18,90 | 4,50 | 59,70 | 1,30 | 45,60 | 35,00 | 92,20 |
| 112 | HEI2 epi | MUE | HEI 2 | Sph | S.fall | emrs | epi | sph | 3,75 | 277,34 | 227,50 | NA | 15,84 | 574,30 | 18,90 | 4,50 | 59,70 | 1,30 | 45,60 | 35,00 | 92,20 |
| 114 | HEI2 epi | MUE | HEI 3 | Sph | S.fall | emrs | epi | sph | 3,75 | 277,34 | 227,50 | NA | 15,84 | 574,30 | 18,90 | 4,50 | 59,70 | 1,30 | 45,60 | 35,00 | 92,20 |
| 116 | HEI1 epi | MUE | HEI 4 | Sph | S.fall | sub | epi | sph | 4,26 | 354,18 | 72,90 | 2,85 | 14,93 | 401,00 | NA | NA | NA | 1,50 | 48,90 | 32,00 | 97,20 |
| 118 | KIE epi | MUE | KIE 1 | Sph | S.mag | emrs | epi | sph | 4,53 | 482,09 | NA | NA | NA | 783,10 | 28,30 | 4,20 | 58,50 | 0,82 | 45,79 | 56,64 | 92,00 |
| 119 | KIE2 epi | MUE | KIE 2 | Sph | S.mag | emrs | epi | sph | 4,53 | 482,09 | NA | NA | NA | 783,10 | 28,30 | 4,20 | 58,50 | 0,82 | 45,79 | 56,64 | 92,00 |
| 121 | KIE3 epi | MUE | KIE 3 | Sph | S.mag | emrs | epi | sph | 4,53 | 482,09 | NA | NA | NA | 783,10 | 28,30 | 4,20 | 58,50 | 0,82 | 45,79 | 56,64 | 92,00 |
| 123 | TW2 epi | SV | TW 2 | Amb | Amb | sub | epi | amb | 5,90 | 0,84 | 1,08 | 6,62 | 11,00 | 430,50 | 48,00 | 1,40 | 56,40 | 1,27 | 28,22 | 22,20 | 51,70 |
| 124 | TW1 epi | SV | TW 1 | Amb | Amb | sub | epi | amb | 5,90 | 0,84 | 1,08 | 6,62 | 11,00 | 430,50 | 48,00 | 1,40 | 56,40 | 1,27 | 28,22 | 22,20 | 51,70 |
| 125 | PC epi | SA | S9 | Amb | Amb | sub | epi | amb | 6,45 | 7,03 | NA | NA | NA | 566,00 | 27,50 | 1,70 | 37,30 | 1,22 | 64,12 | 52,76 | 57,10 |
| 126 | PP1 epi | SA | SuScor a | Amb | Scor | sub | epi | amb | 6,58 | 27,47 | 4,00 | 5,45 | 12,65 | 610,00 | 14,00 | 1,60 | 42,20 | 0,85 | 33,99 | 39,93 | 57,10 |
| 127 | PP1 epi | SA | SuScor b | Amb | Scor | sub | epi | amb | 6,58 | 27,47 | 4,00 | 5,45 | 12,65 | 610,00 | 14,00 | 1,60 | 42,20 | 0,85 | 33,99 | 39,93 | 57,10 |
| 128 | GLU Sed | SV | GLU | Sed | Sed | sub | Ref | amb | 7,00 | 91,53 | NA | NA | 7,20 | NA | NA | NA | NA | NA | NA | NA | NA |
| 129 | GLU Sed | SV | GLU | Sed | Sed | sub | Ref | amb | 7,00 | 91,53 | NA | NA | 7,20 | NA | NA | NA | NA | NA | NA | NA | NA |
| 130 | TW Sed | SV | TW | Sed | Sed | sub | Ref | amb | 5,90 | NA | NA | NA | 10,70 | NA | NA | NA | NA | NA | NA | NA | NA |
| 131 | TW Sed | SV | TW | Sed | Sed | sub | Ref | amb | 5,90 | NA | NA | NA | 10,70 | NA | NA | NA | NA | NA | NA | NA | NA |
| 132 | KLO1 Vasc | MUE | KLO-o 2 | Vasc | Erio | emrs | Ref | sph | 4,15 | 359,64 | 45,30 | 1,64 | 16,50 | NA | 38,60 | 2,00 | 54,20 | 1,03 | 50,47 | 68,31 | NA |
| 133 | KLO1 Vasc | MUE | KLO-o 2 | Vasc | Erio | emrs | Ref | sph | 4,15 | 359,64 | 45,30 | 1,64 | 16,50 | NA | 38,60 | 2,00 | 54,20 | 1,03 | 50,47 | 68,31 | NA |
| 134 | KNU1 endo | SV | KNU 1 | Amb | Amb | sub | endo | amb | 6,25 | 2,76 | 2,55 | 7,63 | 12,05 | NA | NA | NA | NA | NA | NA | NA | 52,30 |
| 135 | KNU1 endo | SV | KNU 1 | Amb | Amb | sub | endo | amb | 6,25 | 2,76 | 2,55 | 7,63 | 12,05 | NA | NA | NA | NA | NA | NA | NA | 52,30 |
| 136 | KNU2 endo | SV | KNU 2 | Amb | Amb | sub | endo | amb | 6,25 | 2,76 | 2,55 | 7,63 | 12,05 | NA | NA | NA | NA | NA | NA | NA | 52,30 |
| 137 | KNU2 endo | SV | KNU 2 | Amb | Amb | sub | endo | amb | 6,25 | 2,76 | 2,55 | 7,63 | 12,05 | NA | NA | NA | NA | NA | NA | NA | 52,30 |
| 138 | PP1 Sed | SA | SuScor | Sed | Sed | sub | Ref | amb | 6,58 | 9,42 | NA | NA | 7,05 | NA | NA | NA | NA | NA | NA | NA | NA |
| 139 | PP2 Sed | SA | SuScor | Sed | Sed | sub | Ref | amb | 6,58 | 9,42 | NA | NA | 7,05 | NA | NA | NA | NA | NA | NA | NA | NA |
| 140 | PP2 Sed | SA | SuCar | Sed | Sed | sub | Ref | amb | 6,58 | 9,42 | NA | NA | 7,05 | NA | NA | NA | NA | NA | NA | NA | NA |
| 141 | PP2 Sed | SA | SuCar | Sed | Sed | sub | Ref | amb | 6,58 | 9,42 | NA | NA | 7,05 | NA | NA | NA | NA | NA | NA | NA | NA |
| 142 | NEI1 Vasc | NE | 1 | Vasc | Erio | emrs | Ref | sph | 3,75 | 151,60 | 59,98 | 3,85 | 12,16 | NA | NA | NA | NA | NA | NA | NA | NA |
| 143 | NEI1 Vasc | NE | 1 | Vasc | Erio | emrs | Ref | sph | 3,75 | 151,60 | 59,98 | 3,85 | 12,16 | NA | NA | NA | NA | NA | NA | NA | NA |
| 144 | NEI5 Cx | NE | 2 | Vasc | Cx | emrs | Ref | sph | 4,63 | 296,92 | 83,98 | 4,32 | 12,45 | NA | NA | NA | NA | NA | NA | NA | NA |
| 145 | NEI5 Cx | NE | 2 | Vasc | Cx | emrs | Ref | sph | 4,63 | 296,92 | 83,98 | 4,32 | 12,45 | NA | NA | NA | NA | NA | NA | NA | NA |
| 146 | PC Sed | SA | S9 | Sed | Sed | sub | Ref | amb | 6,45 | 522,87 | NA | NA | NA | NA | NA | NA | NA | NA | NA | NA | NA |
| 147 | PC Sed | SA | S9 | Sed | Sed | sub | Ref | amb | 6,45 | 522,87 | NA | NA | NA | NA | NA | NA | NA | NA | NA | NA | NA |
| 148 | NEI1 Sed | NE | 1 | Sed | Sed | sub | Ref | sph | 3,75 | 282,62 | NA | NA | 10,99 | NA | NA | NA | NA | NA | NA | NA | NA |
| 149 | NEI1 Sed | NE | 1 | Sed | Sed | sub | Ref | sph | 3,75 | 282,62 | NA | NA | 10,99 | NA | NA | NA | NA | NA | NA | NA | NA |
| 150 | NEI2 Sed | NE | 4 | Sed | Sed | sub | Ref | sph | 4,35 | 979,62 | NA | NA | NA | NA | NA | NA | NA | NA | NA | NA | NA |
| 151 | NEI2 Sed | NE | 4 | Sed | Sed | sub | Ref | sph | 4,35 | 979,62 | NA | NA | NA | NA | NA | NA | NA | NA | NA | NA | NA |
| 152 | NEI4 Sed | NE | 4-2 | Sed | Sed | sub | Ref | sph | 4,35 | 618,29 | NA | NA | NA | NA | NA | NA | NA | NA | NA | NA | NA |
| 153 | NEI4 Sed | NE | 4-2 | Sed | Sed | sub | Ref | sph | 4,95 | 618,29 | NA | NA | NA | NA | NA | NA | NA | NA | NA | NA | NA |
| 154 | NEI3 Sed | NE | 6 | Sed | Sed | sub | Ref | sph | 4,03 | 262,27 | 0,00 | NA | 4,56 | NA | NA | NA | NA | NA | NA | NA | NA |
| 155 | NEI3 Sed | NE | 6 | Sed | Sed | sub | Ref | sph | 4,03 | 262,27 | 0,00 | NA | 4,56 | NA | NA | NA | NA | NA | NA | NA | NA |
| 156 | KNU1 epi | SV | KNU 1 | Amb | Amb | sub | epi | amb | 6,25 | 2,76 | 2,55 | 7,63 | 12,05 | NA | NA | NA | NA | NA | NA | NA | 52,30 |
| 157 | KNU2 epi | SV | KNU 2 | Amb | Amb | sub | epi | amb | 6,25 | 2,76 | 2,55 | 7,63 | 12,05 | NA | NA | NA | NA | NA | NA | NA | 52,30 |

**Supplementary Table S1B:** Coordinates and primer sequences of individual samples

| Sample ID | Name | StudySite | SubSite | Taxon | Taxon-2 | Hydrol | Type | System | Coordinates | Sequence primer F BACTERIA | Sequence primerR BACTERIA | Sequence primer F ARCHAEA | Sequence primerR ARCHAEA |
| --- | --- | --- | --- | --- | --- | --- | --- | --- | --- | --- | --- | --- | --- |
| 1 | GLU1 endo | SV | GLU 1 | Amb | Amb | sub | endo | amb | N78° 32' 48.948" E12° 2' 51.576" | ACACGT CCTACGGGNGGCWGCAG | CAGTCA GACTACHVGGGTATCTAATCC | ACACGT gYg CAS CAg KCg MgA AW | CGATAT GGACTACVSGGGTATCTAAT |
| 2 | GLU1 endo | SV | GLU 1 | Amb | Amb | sub | endo | amb | N78° 32' 48.948" E12° 2' 51.576" | ACACGT CCTACGGGNGGCWGCAG | CATGAC GACTACHVGGGTATCTAATCC | ACACGT gYg CAS CAg KCg MgA AW | CGCGCG GGACTACVSGGGTATCTAAT |
| 3 | TW2 endo | SV | TW 2 | Amb | Amb | sub | endo | amb | N78° 33' 0.18" E11° 31' 39.144" | ACGTAC CCTACGGGNGGCWGCAG | CATGAC GACTACHVGGGTATCTAATCC | ACGTAC gYg CAS CAg KCg MgA AW | CGCGCG GGACTACVSGGGTATCTAAT |
| 4 | TW2 endo | SV | TW 2 | Amb | Amb | sub | endo | amb | N78° 33' 0.18" E11° 31' 39.144" | ACGTAC CCTACGGGNGGCWGCAG | GACTAG GACTACHVGGGTATCTAATCC | ACGTAC gYg CAS CAg KCg MgA AW | CGTATA GGACTACVSGGGTATCTAAT |
| 5 | NEI1 endo | NE | 1 | Sph | S.rip | sub | endo | sph | N69° 24' 36" E29° 6' 36" | ACTGCA CCTACGGGNGGCWGCAG | GACTAG GACTACHVGGGTATCTAATCC | ACTGCA gYg CAS CAg KCg MgA AW | CGTATA GGACTACVSGGGTATCTAAT |
| 6 | NEI1 endo | NE | 1 | Sph | S.rip | sub | endo | sph | N69° 24' 36" E29° 6' 36" | ACTGCA CCTACGGGNGGCWGCAG | GAGATC GACTACHVGGGTATCTAATCC | ATATCG gYg CAS CAg KCg MgA AW | TATACG GGACTACVSGGGTATCTAAT |
| 7 | NEI3 endo | NE | 6 | Sph | S.rip | sub | endo | sph | N69° 24' 38.988" E2° 54' 43.106" | AGCTGA CCTACGGGNGGCWGCAG | GATCGA GACTACHVGGGTATCTAATCC | AGCTGA gYg CAS CAg KCg MgA AW | TAGCAT GGACTACVSGGGTATCTAAT |
| 8 | NEI3 endo | NE | 6 | Sph | S.rip | sub | endo | sph | N69° 24' 38.988" E2° 54' 43.106" | AGCTGA CCTACGGGNGGCWGCAG | GTACAC GACTACHVGGGTATCTAATCC | AGCTGA gYg CAS CAg KCg MgA AW | TATACG GGACTACVSGGGTATCTAAT |
| 9 | GLU2 endo | SV | GLU 2 | Amb | Amb | sub | endo | amb | N78° 32' 48.948" E12° 2' 51.576" | ACACGT CCTACGGGNGGCWGCAG | GAGATC GACTACHVGGGTATCTAATCC | ACACGT gYg CAS CAg KCg MgA AW | TACGTA GGACTACVSGGGTATCTAAT |
| 10 | GLU2 endo | SV | GLU 2 | Amb | Amb | sub | endo | amb | N78° 32' 48.948" E12° 2' 51.576" | ACACGT CCTACGGGNGGCWGCAG | GATCGA GACTACHVGGGTATCTAATCC | ACACGT gYg CAS CAg KCg MgA AW | TAGCAT GGACTACVSGGGTATCTAAT |
| 11 | NEI6 endo | NE | 2-2 | Sph | S.lind | emrs | endo | sph | N69° 24' 40.752" E2° 17' 28.239" | AGAGTC CCTACGGGNGGCWGCAG | GATCGA GACTACHVGGGTATCTAATCC | AGAGTC gYg CAS CAg KCg MgA AW | TAGCAT GGACTACVSGGGTATCTAAT |
| 12 | NEI6 endo | NE | 2-2 | Sph | S.lind | emrs | endo | sph | N69° 24' 40.752" E2° 17' 28.239" | AGAGTC CCTACGGGNGGCWGCAG | GTACAC GACTACHVGGGTATCTAATCC | AGAGTC gYg CAS CAg KCg MgA AW | TATACG GGACTACVSGGGTATCTAAT |
| 13 | NEI4 endo | NE | 4-2 | Sph | S.rip | sub | endo | sph | N69° 24' 38.34" E29° 7' 15.6" | ACACGT CCTACGGGNGGCWGCAG | GTGTGT GACTACHVGGGTATCTAATCC | AGAGTC gYg CAS CAg KCg MgA AW | CGTATA GGACTACVSGGGTATCTAAT |
| 14 | NEI4 endo | NE | 4-2 | Sph | S.rip | sub | endo | sph | N69° 24' 38.34" E29° 7' 15.6" | ACACGT CCTACGGGNGGCWGCAG | TCAGAG GACTACHVGGGTATCTAATCC | AGAGTC gYg CAS CAg KCg MgA AW | TACGTA GGACTACVSGGGTATCTAAT |
| 15 | NEI2 endo | NE | 4 | Sph | S.rip | sub | endo | sph | N69° 24' 36" E29° 7' 12" | ACACGT CCTACGGGNGGCWGCAG | CAGTCA GACTACHVGGGTATCTAATCC | ACTGCA gYg CAS CAg KCg MgA AW | TGCATG GGACTACVSGGGTATCTAAT |
| 16 | NEI2 endo | NE | 4 | Sph | S.rip | sub | endo | sph | N69° 24' 36" E29° 7' 12" | ACACGT CCTACGGGNGGCWGCAG | CATGAC GACTACHVGGGTATCTAATCC | ACTGCA gYg CAS CAg KCg MgA AW | TGACGT GGACTACVSGGGTATCTAAT |
| 17 | NEI5 endo | NE | 2 | Sph | S.lind | emrs | endo | sph | N69° 24' 36" E29° 6' 36" | ACTGCA CCTACGGGNGGCWGCAG | TCGAGA GACTACHVGGGTATCTAATCC | ACTGCA gYg CAS CAg KCg MgA AW | TGTGAC GGACTACVSGGGTATCTAAT |
| 18 | NEI5 endo | NE | 2 | Sph | S.lind | emrs | endo | sph | N69° 24' 36" E29° 6' 36" | AGAGTC CCTACGGGNGGCWGCAG | CAGTCA GACTACHVGGGTATCTAATCC | AGAGTC gYg CAS CAg KCg MgA AW | CGATAT GGACTACVSGGGTATCTAAT |
| 19 | KLO2 endo | MUE | KLO-m 5 | Sph | S.fall | emrs | endo | sph | N53° 12' 21.816" E13° 7' 8.904" | CACAGT CCTACGGGNGGCWGCAG | GAGATC GACTACHVGGGTATCTAATCC | CACAGT gYg CAS CAg KCg MgA AW | TACGTA GGACTACVSGGGTATCTAAT |
| 20 | KLO2 endo | MUE | KLO-m 5 | Sph | S.fall | emrs | endo | sph | N53° 12' 21.816" E13° 7' 8.904" | CACAGT CCTACGGGNGGCWGCAG | GATCGA GACTACHVGGGTATCTAATCC | CACAGT gYg CAS CAg KCg MgA AW | TAGCAT GGACTACVSGGGTATCTAAT |
| 21 | KLO1 endo | MUE | KLO-o 3 | Sph | S.mag | emrs | endo | sph | N53° 12' 21.888" E13° 7' 10.524" | ATGCTA CCTACGGGNGGCWGCAG | GTGTGT GACTACHVGGGTATCTAATCC | ATGCTA gYg CAS CAg KCg MgA AW | TGCATG GGACTACVSGGGTATCTAAT |
| 22 | KLO1 endo | MUE | KLO-o 3 | Sph | S.mag | emrs | endo | sph | N53° 12' 21.888" E13° 7' 10.524" | ATGCTA CCTACGGGNGGCWGCAG | TCAGAG GACTACHVGGGTATCTAATCC | ATGCTA gYg CAS CAg KCg MgA AW | TGACGT GGACTACVSGGGTATCTAAT |
| 23 | HEI2 endo | MUE | HEI 2 | Sph | S.fall | emrs | endo | sph | N53° 12' 8.46" E13° 8' 45.924" | ACGTAC CCTACGGGNGGCWGCAG | TCAGAG GACTACHVGGGTATCTAATCC | AGCTGA gYg CAS CAg KCg MgA AW | TACGTA GGACTACVSGGGTATCTAAT |
| 24 | HEI2 endo | MUE | HEI 2 | Sph | S.fall | emrs | endo | sph | N53° 12' 8.46" E13° 8' 45.924" | ACGTAC CCTACGGGNGGCWGCAG | TCGAGA GACTACHVGGGTATCTAATCC | AGCTGA gYg CAS CAg KCg MgA AW | TAGCAT GGACTACVSGGGTATCTAAT |
| 25 | KIE1 endo | MUE | KIE 1 | Sph | S.mag | emrs | endo | sph | N53° 13' 11.316" E13° 7' 1.992" | ATATCG CCTACGGGNGGCWGCAG | TCAGAG GACTACHVGGGTATCTAATCC | ATATCG gYg CAS CAg KCg MgA AW | TGACGT GGACTACVSGGGTATCTAAT |
| 26 | KIE1 endo | MUE | KIE 1 | Sph | S.mag | emrs | endo | sph | N53° 13' 11.316" E13° 7' 1.992" | ATATCG CCTACGGGNGGCWGCAG | TCGAGA GACTACHVGGGTATCTAATCC | ATATCG gYg CAS CAg KCg MgA AW | TGTGAC GGACTACVSGGGTATCTAAT |
| 27 | KLO2 endo | MUE | KLO-m 4 | Sph | S.fall | emrs | endo | sph | N53° 12' 21.888" E13° 7' 10.524" | CACAGT CCTACGGGNGGCWGCAG | CAGTCA GACTACHVGGGTATCTAATCC | CACAGT gYg CAS CAg KCg MgA AW | CGATAT GGACTACVSGGGTATCTAAT |
| 28 | KLO2 endo | MUE | KLO-m 4 | Sph | S.fall | emrs | endo | sph | N53° 12' 21.888" E13° 7' 10.524" | CACAGT CCTACGGGNGGCWGCAG | CATGAC GACTACHVGGGTATCTAATCC | CACAGT gYg CAS CAg KCg MgA AW | CGCGCG GGACTACVSGGGTATCTAAT |
| 31 | NEI7 endo | NE | 2-3 | Sph | S.lind | emrs | endo | sph | N69° 24' 40.968" E29° 7' 1.812" | AGAGTC CCTACGGGNGGCWGCAG | TCGAGA GACTACHVGGGTATCTAATCC | AGAGTC gYg CAS CAg KCg MgA AW | TGTGAC GGACTACVSGGGTATCTAAT |
| 32 | NEI7 endo | NE | 2-3 | Sph | S.lind | emrs | endo | sph | N69° 24' 40.968" E29° 7' 1.812" | AGCTGA CCTACGGGNGGCWGCAG | CAGTCA GACTACHVGGGTATCTAATCC | AGCTGA gYg CAS CAg KCg MgA AW | CGATAT GGACTACVSGGGTATCTAAT |
| 33 | KLO1 endo | MUE | KLO-o 1 | Sph | S.mag | emrs | endo | sph | N53° 12' 22.896" E13° 7' 10.92" | ATCGAT CCTACGGGNGGCWGCAG | TCGAGA GACTACHVGGGTATCTAATCC | ATCGAT gYg CAS CAg KCg MgA AW | TGTGAC GGACTACVSGGGTATCTAAT |
| 34 | KLO1 endo | MUE | KLO-o 1 | Sph | S.mag | emrs | endo | sph | N53° 12' 22.896" E13° 7' 10.92" | ATGCTA CCTACGGGNGGCWGCAG | CAGTCA GACTACHVGGGTATCTAATCC | ATGCTA gYg CAS CAg KCg MgA AW | CGATAT GGACTACVSGGGTATCTAAT |
| 35 | KLO1 endo | MUE | KLO-o 2 | Sph | S.mag | emrs | endo | sph | N53° 12' 22.392" E13° 7' 10.2" | ATGCTA CCTACGGGNGGCWGCAG | GACTAG GACTACHVGGGTATCTAATCC | ATGCTA gYg CAS CAg KCg MgA AW | CGTATA GGACTACVSGGGTATCTAAT |
| 36 | KLO1 endo | MUE | KLO-o 2 | Sph | S.mag | emrs | endo | sph | N53° 12' 22.392" E13° 7' 10.2" | ATGCTA CCTACGGGNGGCWGCAG | GAGATC GACTACHVGGGTATCTAATCC | ATGCTA gYg CAS CAg KCg MgA AW | TACGTA GGACTACVSGGGTATCTAAT |
| 37 | KLO2 endo | MUE | KLO-m 6 | Sph | S.fall | emrs | endo | sph | N53° 12' 22.716" E13° 7' 9.516" | CACAGT CCTACGGGNGGCWGCAG | GTCACA GACTACHVGGGTATCTAATCC | CACAGT gYg CAS CAg KCg MgA AW | TCTCTC GGACTACVSGGGTATCTAAT |
| 38 | KLO2 endo | MUE | KLO-m 6 | Sph | S.fall | emrs | endo | sph | N53° 12' 22.716" E13° 7' 9.516" | CACAGT CCTACGGGNGGCWGCAG | GTGTGT GACTACHVGGGTATCTAATCC | CACAGT gYg CAS CAg KCg MgA AW | TGCATG GGACTACVSGGGTATCTAAT |
| 41 | KIE2 endo | MUE | KIE 2 | Sph | S.mag | emrs | endo | sph | N53° 13' 11.82" E13° 7' 1.956" | ATCGAT CCTACGGGNGGCWGCAG | GAGATC GACTACHVGGGTATCTAATCC | ATCGAT gYg CAS CAg KCg MgA AW | TACGTA GGACTACVSGGGTATCTAAT |
| 45 | TW1 endo | SV | TW 1 | Amb | Amb | sub | endo | amb | N78° 33' 0.18" E11° 31' 39.144" | ACACGT CCTACGGGNGGCWGCAG | TCAGAG GACTACHVGGGTATCTAATCC | ACACGT gYg CAS CAg KCg MgA AW | TGACGT GGACTACVSGGGTATCTAAT |
| 46 | TW1 endo | SV | TW 1 | Amb | Amb | sub | endo | amb | N78° 33' 0.18" E11° 31' 39.144" | ACACGT CCTACGGGNGGCWGCAG | TCGAGA GACTACHVGGGTATCTAATCC | ACACGT gYg CAS CAg KCg MgA AW | TGTGAC GGACTACVSGGGTATCTAAT |
| 47 | KIE3 endo | MUE | KIE 3 | Sph | S.mag | emrs | endo | sph | N53° 13' 11.604" E13° 7' 1.884" | ATCGAT CCTACGGGNGGCWGCAG | GTCACA GACTACHVGGGTATCTAATCC | ATCGAT gYg CAS CAg KCg MgA AW | TCTCTC GGACTACVSGGGTATCTAAT |
| 48 | KIE3 endo | MUE | KIE 3 | Sph | S.mag | emrs | endo | sph | N53° 13' 11.604" E13° 7' 1.884" | ATCGAT CCTACGGGNGGCWGCAG | GTGTGT GACTACHVGGGTATCTAATCC | ATCGAT gYg CAS CAg KCg MgA AW | TGCATG GGACTACVSGGGTATCTAAT |
| 49 | PP1 endo | SA | SuScor a | Amb | Scor | sub | endo | amb | N72° 22' 11.82" E12° 38' 53.722" | AGTCAG CCTACGGGNGGCWGCAG | CAGTCA GACTACHVGGGTATCTAATCC | AGTCAG gYg CAS CAg KCg MgA AW | CGATAT GGACTACVSGGGTATCTAAT |
| 50 | PP1 endo | SA | SuScor a | Amb | Scor | sub | endo | amb | N72° 22' 11.82" E12° 38' 53.722" | AGTCAG CCTACGGGNGGCWGCAG | CATGAC GACTACHVGGGTATCTAATCC | AGTCAG gYg CAS CAg KCg MgA AW | CGCGCG GGACTACVSGGGTATCTAAT |
| 51 | PP1 endo | SA | SuScor b | Amb | Scor | sub | endo | amb | N72° 22' 11.82" E12° 38' 53.722" | AGTCAG CCTACGGGNGGCWGCAG | GAGATC GACTACHVGGGTATCTAATCC | AGTCAG gYg CAS CAg KCg MgA AW | TACGTA GGACTACVSGGGTATCTAAT |
| 52 | PP1 endo | SA | SuScor b | Amb | Scor | sub | endo | amb | N72° 22' 11.82" E12° 38' 53.722" | AGTCAG CCTACGGGNGGCWGCAG | GATCGA GACTACHVGGGTATCTAATCC | AGTCAG gYg CAS CAg KCg MgA AW | TAGCAT GGACTACVSGGGTATCTAAT |
| 53 | PC endo | SA | S9 | Amb | Amb | sub | endo | amb | N72° 22' 16.104" E12° 38' 56.4" | ATATCG CCTACGGGNGGCWGCAG | GAGATC GACTACHVGGGTATCTAATCC | ATATCG gYg CAS CAg KCg MgA AW | TACGTA GGACTACVSGGGTATCTAAT |
| 54 | PC endo | SA | S9 | Amb | Amb | sub | endo | amb | N72° 22' 16.104" E12° 38' 56.4" | ATATCG CCTACGGGNGGCWGCAG | GATCGA GACTACHVGGGTATCTAATCC | ATATCG gYg CAS CAg KCg MgA AW | TAGCAT GGACTACVSGGGTATCTAAT |
| 55 | HEI2 endo | MUE | HEI 1 | Sph | S.fall | emrs | endo | sph | N53° 12' 8.568" E13° 8' 45.996" | ACGTAC CCTACGGGNGGCWGCAG | GACTAG GACTACHVGGGTATCTAATCC | AGAGTC gYg CAS CAg KCg MgA AW | TGACGT GGACTACVSGGGTATCTAAT |
| 56 | HEI2 endo | MUE | HEI 1 | Sph | S.fall | emrs | endo | sph | N53° 12' 8.568" E13° 8' 45.996" | ACGTAC CCTACGGGNGGCWGCAG | GATCGA GACTACHVGGGTATCTAATCC | AGAGTC gYg CAS CAg KCg MgA AW | TGTGAC GGACTACVSGGGTATCTAAT |
| 57 | HEI2 endo | MUE | HEI 3 | Sph | S.fall | emrs | endo | sph | N53° 12' 8.136" E13° 8' 47.004" | ACTGCA CCTACGGGNGGCWGCAG | CATGAC GACTACHVGGGTATCTAATCC | AGTCAG gYg CAS CAg KCg MgA AW | CGTATA GGACTACVSGGGTATCTAAT |
| 58 | HEI2 endo | MUE | HEI 3 | Sph | S.fall | emrs | endo | sph | N53° 12' 8.136" E13° 8' 47.004" | ACTGCA CCTACGGGNGGCWGCAG | GACTAG GACTACHVGGGTATCTAATCC | AGCTGA gYg CAS CAg KCg MgA AW | TGCATG GGACTACVSGGGTATCTAAT |
| 59 | HEI1 endo | MUE | HEI 4 | Sph | S.fall | sub | endo | sph | N53° 12' 7.128" E13° 8' 46.32" | ACTGCA CCTACGGGNGGCWGCAG | GATCGA GACTACHVGGGTATCTAATCC | AGCTGA gYg CAS CAg KCg MgA AW | TGTGAC GGACTACVSGGGTATCTAAT |
| 60 | HEI1 endo | MUE | HEI 4 | Sph | S.fall | sub | endo | sph | N53° 12' 7.128" E13° 8' 46.32" | ACTGCA CCTACGGGNGGCWGCAG | GTACAC GACTACHVGGGTATCTAATCC | AGTCAG gYg CAS CAg KCg MgA AW | CGATAT GGACTACVSGGGTATCTAAT |
| 67 | KLO2 Vasc | MUE | KLO-m 6 | Vasc | Cx | emrs | Ref | sph | N53° 12' 22.716" E13° 7' 9.516" | CACAGT CCTACGGGNGGCWGCAG | TCAGAG GACTACHVGGGTATCTAATCC | CACAGT gYg CAS CAg KCg MgA AW | TGACGT GGACTACVSGGGTATCTAAT |
| 68 | KLO2 Vasc | MUE | KLO-m 6 | Vasc | Cx | emrs | Ref | sph | N53° 12' 22.716" E13° 7' 9.516" | CACAGT CCTACGGGNGGCWGCAG | TCGAGA GACTACHVGGGTATCTAATCC | CACAGT gYg CAS CAg KCg MgA AW | TGTGAC GGACTACVSGGGTATCTAAT |
| 69 | NEI7 Vasc | NE | 2-3 | Vasc | Vasc | emrs | Ref | sph | N69° 24' 40.968" E29° 7' 1.812" | AGCTGA CCTACGGGNGGCWGCAG | GACTAG GACTACHVGGGTATCTAATCC | AGCTGA gYg CAS CAg KCg MgA AW | TGTGAC GGACTACVSGGGTATCTAAT |
| 70 | NEI7 Vasc | NE | 2-3 | Vasc | Vasc | emrs | Ref | sph | N69° 24' 40.968" E29° 7' 1.812" | AGCTGA CCTACGGGNGGCWGCAG | GAGATC GACTACHVGGGTATCTAATCC | AGCTGA gYg CAS CAg KCg MgA AW | TACGTA GGACTACVSGGGTATCTAAT |
| 71 | KIE Vasc | MUE | KIE 1 | Vasc | Cx | emrs | Ref | sph | N53° 13' 11.316" E13° 7' 1.992" | ATCGAT CCTACGGGNGGCWGCAG | CAGTCA GACTACHVGGGTATCTAATCC | ATCGAT gYg CAS CAg KCg MgA AW | CGATAT GGACTACVSGGGTATCTAAT |
| 72 | KIE Vasc | MUE | KIE 1 | Vasc | Cx | emrs | Ref | sph | N53° 13' 11.316" E13° 7' 1.992" | ATCGAT CCTACGGGNGGCWGCAG | CATGAC GACTACHVGGGTATCTAATCC | ATCGAT gYg CAS CAg KCg MgA AW | CGCGCG GGACTACVSGGGTATCTAAT |
| 73 | NEI6 Vasc | NE | 2-2 | Vasc | Cx | emrs | Ref | sph | N69° 24' 40.752" E2° 17' 28.239" | AGAGTC CCTACGGGNGGCWGCAG | GTGTGT GACTACHVGGGTATCTAATCC | AGAGTC gYg CAS CAg KCg MgA AW | TGCATG GGACTACVSGGGTATCTAAT |
| 74 | NEI6 Vasc | NE | 2-2 | Vasc | Cx | emrs | Ref | sph | N69° 24' 40.752" E2° 17' 28.239" | AGAGTC CCTACGGGNGGCWGCAG | TCAGAG GACTACHVGGGTATCTAATCC | AGAGTC gYg CAS CAg KCg MgA AW | TGACGT GGACTACVSGGGTATCTAAT |
| 75 | PP2 endo | SA | SuCar | Amb | Scor | sub | endo | amb | N72° 22' 11.82" E12° 38' 53.722" | AGTCAG CCTACGGGNGGCWGCAG | TCAGAG GACTACHVGGGTATCTAATCC | AGTCAG gYg CAS CAg KCg MgA AW | TGACGT GGACTACVSGGGTATCTAAT |
| 76 | PP2 endo | SA | SuCar | Amb | Scor | sub | endo | amb | N72° 22' 11.82" E12° 38' 53.722" | AGTCAG CCTACGGGNGGCWGCAG | TCGAGA GACTACHVGGGTATCTAATCC | AGTCAG gYg CAS CAg KCg MgA AW | TGTGAC GGACTACVSGGGTATCTAAT |
| 77 | HEI2 Vasc | MUE | HEI 1 | Vasc | Erio | emrs | Ref | sph | N53° 12' 8.568" E13° 8' 45.996" | ACGTAC CCTACGGGNGGCWGCAG | GTCACA GACTACHVGGGTATCTAATCC | AGCTGA gYg CAS CAg KCg MgA AW | CGCGCG GGACTACVSGGGTATCTAAT |
| 78 | HEI2 Vasc | MUE | HEI 1 | Vasc | Erio | emrs | Ref | sph | N53° 12' 8.568" E13° 8' 45.996" | ACGTAC CCTACGGGNGGCWGCAG | GTGTGT GACTACHVGGGTATCTAATCC | AGCTGA gYg CAS CAg KCg MgA AW | CGTATA GGACTACVSGGGTATCTAAT |
| 86 | PP2 epi | SA | SuCar | Amb | Scor | sub | epi | amb | N72° 22' 11.82" E12° 38' 53.722" | ATATCG CCTACGGGNGGCWGCAG | CAGTCA GACTACHVGGGTATCTAATCC | ATATCG gYg CAS CAg KCg MgA AW | CGATAT GGACTACVSGGGTATCTAAT |
| 89 | KLO1 epi | MUE | KLO-o 1 | Sph | S.mag | emrs | epi | sph | N53° 12' 22.896" E13° 7' 10.92" | ATGCTA CCTACGGGNGGCWGCAG | CATGAC GACTACHVGGGTATCTAATCC | ATGCTA gYg CAS CAg KCg MgA AW | CGCGCG GGACTACVSGGGTATCTAAT |
| 90 | KLO1 epi | MUE | KLO-o 2 | Sph | S.mag | emrs | epi | sph | N53° 12' 22.392" E13° 7' 10.2" | ATGCTA CCTACGGGNGGCWGCAG | GATCGA GACTACHVGGGTATCTAATCC | ATGCTA gYg CAS CAg KCg MgA AW | TAGCAT GGACTACVSGGGTATCTAAT |
| 92 | KLO2 epi | MUE | KLO-m 3 | Sph | S.fall | emrs | epi | sph | N53° 12' 21.888" E13° 7' 10.524" | ATGCTA CCTACGGGNGGCWGCAG | TCGAGA GACTACHVGGGTATCTAATCC | ATGCTA gYg CAS CAg KCg MgA AW | TGTGAC GGACTACVSGGGTATCTAAT |
| 93 | KLO2 epi | MUE | KLO-m 4 | Sph | S.fall | emrs | epi | sph | N53° 12' 21.888" E13° 7' 10.524" | CACAGT CCTACGGGNGGCWGCAG | GACTAG GACTACHVGGGTATCTAATCC | CACAGT gYg CAS CAg KCg MgA AW | CGTATA GGACTACVSGGGTATCTAAT |
| 94 | KLO2 epi | MUE | KLO-m 5 | Sph | S.fall | emrs | epi | sph | N53° 12' 21.816" E13° 7' 8.904" | CACAGT CCTACGGGNGGCWGCAG | GTACAC GACTACHVGGGTATCTAATCC | CACAGT gYg CAS CAg KCg MgA AW | TATACG GGACTACVSGGGTATCTAAT |
| 98 | NEI1 epi | NE | 1 | Sph | S.rip | sub | epi | sph | N69° 24' 36" E29° 6' 36" | ACTGCA CCTACGGGNGGCWGCAG | GATCGA GACTACHVGGGTATCTAATCC | ACTGCA gYg CAS CAg KCg MgA AW | TAGCAT GGACTACVSGGGTATCTAAT |
| 99 | NEI5 epi | NE | 2 | Sph | S.lind | emrs | epi | sph | N69° 24' 36" E29° 6' 36" | AGAGTC CCTACGGGNGGCWGCAG | CATGAC GACTACHVGGGTATCTAATCC | AGAGTC gYg CAS CAg KCg MgA AW | CGCGCG GGACTACVSGGGTATCTAAT |
| 101 | NEI6 epi | NE | 2-2 | Sph | S.lind | emrs | epi | sph | N69° 24' 40.752" E2° 17' 28.239" | ATATCG CCTACGGGNGGCWGCAG | GTCACA GACTACHVGGGTATCTAATCC | ATATCG gYg CAS CAg KCg MgA AW | TGCATG GGACTACVSGGGTATCTAAT |
| 102 | NEI7 epi | NE | 2-3 | Sph | S.rip | emrs | epi | sph | N69° 24' 40.968" E29° 7' 1.812" | AGCTGA CCTACGGGNGGCWGCAG | CATGAC GACTACHVGGGTATCTAATCC | AGCTGA gYg CAS CAg KCg MgA AW | CGCGCG GGACTACVSGGGTATCTAAT |
| 103 | NEI2 epi | NE | 4 | Sph | S.rip | sub | epi | sph | N69° 24' 36" E29° 7' 12" | ACACGT CCTACGGGNGGCWGCAG | GAGATC GACTACHVGGGTATCTAATCC | ACTGCA gYg CAS CAg KCg MgA AW | TGTGAC GGACTACVSGGGTATCTAAT |
| 105 | NEI4 epi | NE | 4-2 | Sph | S.rip | sub | epi | sph | N69° 24' 38.34" E29° 7' 15.6" | ACACGT CCTACGGGNGGCWGCAG | TCGAGA GACTACHVGGGTATCTAATCC | AGAGTC gYg CAS CAg KCg MgA AW | TAGCAT GGACTACVSGGGTATCTAAT |
| 106 | NEI3 epi | NE | 6 | Sph | S.rip | sub | epi | sph | N69° 24' 38.988" E2° 54' 43.106" | AGCTGA CCTACGGGNGGCWGCAG | GTCACA GACTACHVGGGTATCTAATCC | AGCTGA gYg CAS CAg KCg MgA AW | TCTCTC GGACTACVSGGGTATCTAAT |
| 107 | GLU1 epi | SV | GLU 1 | Amb | Amb | sub | epi | amb | N78° 32' 48.948" E12° 2' 51.576" | ATATCG CCTACGGGNGGCWGCAG | CAGTCA GACTACHVGGGTATCTAATCC | ATATCG gYg CAS CAg KCg MgA AW | CGCGCG GGACTACVSGGGTATCTAAT |
| 109 | GLU2 epi | SV | GLU 2 | Amb | Amb | sub | epi | amb | N78° 32' 48.948" E12° 2' 51.576" | ACACGT CCTACGGGNGGCWGCAG | GTACAC GACTACHVGGGTATCTAATCC | ACACGT gYg CAS CAg KCg MgA AW | TATACG GGACTACVSGGGTATCTAAT |
| 111 | HEI2 epi | MUE | HEI 1 | Sph | S.fall | emrs | epi | sph | N53° 12' 8.568" E13° 8' 45.996" | ACGTAC CCTACGGGNGGCWGCAG | GTACAC GACTACHVGGGTATCTAATCC | AGCTGA gYg CAS CAg KCg MgA AW | CGATAT GGACTACVSGGGTATCTAAT |
| 112 | HEI2 epi | MUE | HEI 2 | Sph | S.fall | emrs | epi | sph | N53° 12' 8.46" E13° 8' 45.924" | ACTGCA CCTACGGGNGGCWGCAG | CAGTCA GACTACHVGGGTATCTAATCC | AGCTGA gYg CAS CAg KCg MgA AW | TATACG GGACTACVSGGGTATCTAAT |
| 114 | HEI2 epi | MUE | HEI 3 | Sph | S.fall | emrs | epi | sph | N53° 12' 8.136" E13° 8' 47.004" | ACTGCA CCTACGGGNGGCWGCAG | GAGATC GACTACHVGGGTATCTAATCC | AGCTGA gYg CAS CAg KCg MgA AW | TGACGT GGACTACVSGGGTATCTAAT |
| 116 | HEI1 epi | MUE | HEI 4 | Sph | S.fall | sub | epi | sph | N53° 12' 7.128" E13° 8' 46.32" | ACTGCA CCTACGGGNGGCWGCAG | GTCACA GACTACHVGGGTATCTAATCC | AGTCAG gYg CAS CAg KCg MgA AW | CGCGCG GGACTACVSGGGTATCTAAT |
| 118 | KIE epi | MUE | KIE 1 | Sph | S.mag | emrs | epi | sph | N53° 13' 11.316" E13° 7' 1.992" | ATCGAT CCTACGGGNGGCWGCAG | GACTAG GACTACHVGGGTATCTAATCC | ATCGAT gYg CAS CAg KCg MgA AW | CGTATA GGACTACVSGGGTATCTAAT |
| 119 | KIE2 epi | MUE | KIE 2 | Sph | S.mag | emrs | epi | sph | N53° 13' 11.82" E13° 7' 1.956" | ATCGAT CCTACGGGNGGCWGCAG | GTACAC GACTACHVGGGTATCTAATCC | ATCGAT gYg CAS CAg KCg MgA AW | TATACG GGACTACVSGGGTATCTAAT |
| 121 | KIE3 endo | MUE | KIE 3 | Sph | S.mag | emrs | epi | sph | N53° 13' 11.604" E13° 7' 1.884" | ATCGAT CCTACGGGNGGCWGCAG | TCAGAG GACTACHVGGGTATCTAATCC | ATCGAT gYg CAS CAg KCg MgA AW | TGACGT GGACTACVSGGGTATCTAAT |
| 123 | TW2 epi | SV | TW 2 | Amb | Amb | sub | epi | amb | N78° 33' 0.18" E11° 31' 39.144" | ACGTAC CCTACGGGNGGCWGCAG | GAGATC GACTACHVGGGTATCTAATCC | ACGTAC gYg CAS CAg KCg MgA AW | TACGTA GGACTACVSGGGTATCTAAT |
| 124 | TW1 epi | SV | TW 1 | Amb | Amb | sub | epi | amb | N78° 33' 0.18" E11° 31' 39.144" | ACGTAC CCTACGGGNGGCWGCAG | CAGTCA GACTACHVGGGTATCTAATCC | ACGTAC gYg CAS CAg KCg MgA AW | CGATAT GGACTACVSGGGTATCTAAT |
| 125 | PC epi | SA | S9 | Amb | Amb | sub | epi | amb | N72° 22' 16.104" E12° 38' 56.4" | ATATCG CCTACGGGNGGCWGCAG | GTACAC GACTACHVGGGTATCTAATCC | ATATCG gYg CAS CAg KCg MgA AW | TATACG GGACTACVSGGGTATCTAAT |
| 126 | PP1 epi | SA | SuScor a | Amb | Scor | sub | epi | amb | N72° 22' 11.82" E12° 38' 53.722" | AGTCAG CCTACGGGNGGCWGCAG | GACTAG GACTACHVGGGTATCTAATCC | AGTCAG gYg CAS CAg KCg MgA AW | CGTATA GGACTACVSGGGTATCTAAT |
| 127 | PP1 epi | SA | SuScor b | Amb | Scor | sub | epi | amb | N72° 22' 11.82" E12° 38' 53.722" | AGTCAG CCTACGGGNGGCWGCAG | GTACAC GACTACHVGGGTATCTAATCC | AGTCAG gYg CAS CAg KCg MgA AW | TATACG GGACTACVSGGGTATCTAAT |
| 128 | GLU Sed | SV | GLU | Sed | Sed | sub | Ref | amb | N78° 32' 48.948" E12° 2' 51.576" | ACACGT CCTACGGGNGGCWGCAG | GTCACA GACTACHVGGGTATCTAATCC | ACACGT gYg CAS CAg KCg MgA AW | TCTCTC GGACTACVSGGGTATCTAAT |
| 129 | GLU Sed | SV | GLU | Sed | Sed | sub | Ref | amb | N78° 32' 48.948" E12° 2' 51.576" | ACACGT CCTACGGGNGGCWGCAG | GTGTGT GACTACHVGGGTATCTAATCC | ACACGT gYg CAS CAg KCg MgA AW | TGCATG GGACTACVSGGGTATCTAAT |
| 130 | TW Sed | SV | TW | Sed | Sed | sub | Ref | amb | N78° 33' 0.18" E11° 31' 39.144" | ACGTAC CCTACGGGNGGCWGCAG | GATCGA GACTACHVGGGTATCTAATCC | ATATCG gYg CAS CAg KCg MgA AW | CGTATA GGACTACVSGGGTATCTAAT |
| 131 | TW Sed | SV | TW | Sed | Sed | sub | Ref | amb | N78° 33' 0.18" E11° 31' 39.144" | ACGTAC CCTACGGGNGGCWGCAG | GTACAC GACTACHVGGGTATCTAATCC | ATATCG gYg CAS CAg KCg MgA AW | TACGTA GGACTACVSGGGTATCTAAT |
| 132 | KLO1 Vasc | MUE | KLO-o 2 | Vasc | Erio | emrs | Ref | sph | N53° 12' 22.392" E13° 7' 10.2" | ATATCG CCTACGGGNGGCWGCAG | TCAGAG GACTACHVGGGTATCTAATCC | ATATCG gYg CAS CAg KCg MgA AW | TGTGAC GGACTACVSGGGTATCTAAT |
| 133 | KLO1 Vasc | MUE | KLO-o 2 | Vasc | Erio | emrs | Ref | sph | N53° 12' 22.392" E13° 7' 10.2" | ATGCTA CCTACGGGNGGCWGCAG | GTCACA GACTACHVGGGTATCTAATCC | ATGCTA gYg CAS CAg KCg MgA AW | TCTCTC GGACTACVSGGGTATCTAAT |
| 134 | KNU1 endo | SV | KNU 1 | Amb | Amb | sub | endo | amb | N78° 33' 55.584" E11° 29' 25.98" | ACGTAC CCTACGGGNGGCWGCAG | GTCACA GACTACHVGGGTATCTAATCC | ATATCG gYg CAS CAg KCg MgA AW | TAGCAT GGACTACVSGGGTATCTAAT |
| 135 | KNU1 endo | SV | KNU 1 | Amb | Amb | sub | endo | amb | N78° 33' 55.584" E11° 29' 25.98" | ACGTAC CCTACGGGNGGCWGCAG | GTGTGT GACTACHVGGGTATCTAATCC | ACGTAC gYg CAS CAg KCg MgA AW | TGCATG GGACTACVSGGGTATCTAAT |
| 136 | KNU2 endo | SV | KNU 2 | Amb | Amb | sub | endo | amb | N78° 33' 55.584" E11° 29' 25.98" | ACGTAC CCTACGGGNGGCWGCAG | TCGAGA GACTACHVGGGTATCTAATCC | ACGTAC gYg CAS CAg KCg MgA AW | TGTGAC GGACTACVSGGGTATCTAAT |
| 137 | KNU2 endo | SV | KNU 2 | Amb | Amb | sub | endo | amb | N78° 33' 55.584" E11° 29' 25.98" | ACTGCA CCTACGGGNGGCWGCAG | CAGTCA GACTACHVGGGTATCTAATCC | ACTGCA gYg CAS CAg KCg MgA AW | CGATAT GGACTACVSGGGTATCTAAT |
| 138 | PP1 Sed | SA | SuScor | Sed | Sed | sub | Ref | amb | N72° 22' 11.82" E12° 38' 53.722" | AGTCAG CCTACGGGNGGCWGCAG | GTCACA GACTACHVGGGTATCTAATCC | AGTCAG gYg CAS CAg KCg MgA AW | TCTCTC GGACTACVSGGGTATCTAAT |
| 139 | PP2 Sed | SA | SuScor | Sed | Sed | sub | Ref | amb | N72° 22' 11.82" E12° 38' 53.722" | AGTCAG CCTACGGGNGGCWGCAG | GTGTGT GACTACHVGGGTATCTAATCC | AGTCAG gYg CAS CAg KCg MgA AW | TGCATG GGACTACVSGGGTATCTAAT |
| 140 | PP2 Sed | SA | SuCar | Sed | Sed | sub | Ref | amb | N72° 22' 11.82" E12° 38' 53.722" | ATATCG CCTACGGGNGGCWGCAG | CATGAC GACTACHVGGGTATCTAATCC | ATATCG gYg CAS CAg KCg MgA AW | CGCGCG GGACTACVSGGGTATCTAAT |
| 141 | PP2 Sed | SA | SuCar | Sed | Sed | sub | Ref | amb | N72° 22' 11.82" E12° 38' 53.722" | ATATCG CCTACGGGNGGCWGCAG | GACTAG GACTACHVGGGTATCTAATCC | ATATCG gYg CAS CAg KCg MgA AW | CGTATA GGACTACVSGGGTATCTAAT |
| 142 | NEI1 Vasc | NE | 1 | Vasc | Erio | emrs | Ref | sph | N69° 24' 36" E29° 6' 36" | ACTGCA CCTACGGGNGGCWGCAG | GTACAC GACTACHVGGGTATCTAATCC | ACTGCA gYg CAS CAg KCg MgA AW | TATACG GGACTACVSGGGTATCTAAT |
| 143 | NEI1 Vasc | NE | 1 | Vasc | Erio | emrs | Ref | sph | N69° 24' 36" E29° 6' 36" | ACTGCA CCTACGGGNGGCWGCAG | GTCACA GACTACHVGGGTATCTAATCC | ACTGCA gYg CAS CAg KCg MgA AW | TCTCTC GGACTACVSGGGTATCTAAT |
| 144 | NEI5 Cx | NE | 2 | Vasc | Cx | emrs | Ref | sph | N69° 24' 36" E29° 6' 36" | AGAGTC CCTACGGGNGGCWGCAG | GACTAG GACTACHVGGGTATCTAATCC | AGAGTC gYg CAS CAg KCg MgA AW | CGTATA GGACTACVSGGGTATCTAAT |
| 145 | NEI5 Cx | NE | 2 | Vasc | Cx | emrs | Ref | sph | N69° 24' 36" E29° 6' 36" | AGAGTC CCTACGGGNGGCWGCAG | GAGATC GACTACHVGGGTATCTAATCC | AGAGTC gYg CAS CAg KCg MgA AW | TACGTA GGACTACVSGGGTATCTAAT |
| 146 | PC Sed | SA | S9 | Sed | Sed | sub | Ref | amb | N72° 22' 16.104" E12° 38' 56.4" | ATATCG CCTACGGGNGGCWGCAG | GTCACA GACTACHVGGGTATCTAATCC | ATATCG gYg CAS CAg KCg MgA AW | TCTCTC GGACTACVSGGGTATCTAAT |
| 147 | PC Sed | SA | S9 | Sed | Sed | sub | Ref | amb | N72° 22' 16.104" E12° 38' 56.4" | ATATCG CCTACGGGNGGCWGCAG | GTGTGT GACTACHVGGGTATCTAATCC | ATATCG gYg CAS CAg KCg MgA AW | TGCATG GGACTACVSGGGTATCTAAT |
| 148 | NEI1 Sed | NE | 1 | Sed | Sed | sub | Ref | sph | N69° 24' 36" E29° 6' 36" | ACTGCA CCTACGGGNGGCWGCAG | GTGTGT GACTACHVGGGTATCTAATCC | ACTGCA gYg CAS CAg KCg MgA AW | TGCATG GGACTACVSGGGTATCTAAT |
| 149 | NEI1 Sed | NE | 1 | Sed | Sed | sub | Ref | sph | N69° 24' 36" E29° 6' 36" | ACTGCA CCTACGGGNGGCWGCAG | TCAGAG GACTACHVGGGTATCTAATCC | ACTGCA gYg CAS CAg KCg MgA AW | TGACGT GGACTACVSGGGTATCTAAT |
| 150 | NEI2 Sed | NE | 4 | Sed | Sed | sub | Ref | sph | N69° 24' 36" E29° 7' 12" | ACACGT CCTACGGGNGGCWGCAG | GATCGA GACTACHVGGGTATCTAATCC | AGAGTC gYg CAS CAg KCg MgA AW | CGATAT GGACTACVSGGGTATCTAAT |
| 151 | NEI2 Sed | NE | 4 | Sed | Sed | sub | Ref | sph | N69° 24' 36" E29° 7' 12" | ACACGT CCTACGGGNGGCWGCAG | GTCACA GACTACHVGGGTATCTAATCC | AGAGTC gYg CAS CAg KCg MgA AW | CGCGCG GGACTACVSGGGTATCTAAT |
| 152 | NEI4 Sed | NE | 4-2 | Sed | Sed | sub | Ref | sph | N69° 24' 38.34" E29° 7' 15.6" | ACGTAC CCTACGGGNGGCWGCAG | CAGTCA GACTACHVGGGTATCTAATCC | AGAGTC gYg CAS CAg KCg MgA AW | TATACG GGACTACVSGGGTATCTAAT |
| 153 | NEI4 Sed | NE | 4-2 | Sed | Sed | sub | Ref | sph | N69° 24' 38.34" E29° 7' 15.6" | ACGTAC CCTACGGGNGGCWGCAG | CATGAC GACTACHVGGGTATCTAATCC | AGAGTC gYg CAS CAg KCg MgA AW | TGCATG GGACTACVSGGGTATCTAAT |
| 154 | NEI3 Sed | NE | 6 | Sed | Sed | sub | Ref | sph | N69° 24' 38.988" E2° 54' 43.106" | AGCTGA CCTACGGGNGGCWGCAG | GTGTGT GACTACHVGGGTATCTAATCC | AGCTGA gYg CAS CAg KCg MgA AW | TGCATG GGACTACVSGGGTATCTAAT |
| 155 | NEI3 Sed | NE | 6 | Sed | Sed | sub | Ref | sph | N69° 24' 38.988" E2° 54' 43.106" | AGCTGA CCTACGGGNGGCWGCAG | TCAGAG GACTACHVGGGTATCTAATCC | AGCTGA gYg CAS CAg KCg MgA AW | TGACGT GGACTACVSGGGTATCTAAT |
| 156 | KNU1 epi | SV | KNU 1 | Amb | Amb | sub | epi | amb | N78° 32' 48.948" E12° 2' 51.576" | ACGTAC CCTACGGGNGGCWGCAG | TCAGAG GACTACHVGGGTATCTAATCC | ACGTAC gYg CAS CAg KCg MgA AW | TGACGT GGACTACVSGGGTATCTAAT |
| 157 | KNU2 epi | SV | KNU 2 | Amb | Amb | sub | epi | amb | N78° 32' 48.948" E12° 2' 51.576" | ACTGCA CCTACGGGNGGCWGCAG | CATGAC GACTACHVGGGTATCTAATCC | ACTGCA gYg CAS CAg KCg MgA AW | CGCGCG GGACTACVSGGGTATCTAAT |

**Supplementary Table S2:** Core microbiomes of Amblystegiaceae and *Sphagnum* ecosystems (A), different moss types within these (B) and intersects between the moss core microbiomes (C). Letters A-F in (C) refers to letters A-F in (B) column one.

| **S2 A** | OTU total | OTU core | Number of Amplicon libraries | Threshold for core microbiome calculation | Contribution to relative abundance |
| --- | --- | --- | --- | --- | --- |
| Total ecosystem | 13799 | 49 | 122 | 66 % | 1 - 65% |
| Total moss | 10930 | 52 | 88 | 66 % | 2 - 60% |
| *Sphagnum* ecosystem | 7197 | 119 | 82 | 66 % | 16 - 83% |
| *Sphagnum* moss | 5669 | 142 | 58 | 66 % | 22 - 84% |
| Amblystegiaceae ecosystem | 7612 | 322 | 40 | 66 % | 20 - 77% |
| Amblystegiaceae moss | 6057 | 348 | 30 | 66 % | 44 - 78% |

| **S2 B** | Site | OTU total | OTU core | Number of Amplicon libraries | Threshold for core microbiome calculation | Contribution to relative abundance |
| --- | --- | --- | --- | --- | --- | --- |
| A - Mosses svalbard | SV | 4681 | 295 | 18 | 75 % | 44 - 76% |
| B - Scorpidium | SA | 3022 | 548 | 12 | 75 % | 66 - 83% |
| C - Sphagnum riparium | NE | 1905 | 126 | 12 | 75 % | 36 - 88% |
| D - Sphagum fallax | MUE | 2689 | 132 | 20 | 75 % | 34 - 84% |
| E - Sphagnum lindbergii | NE | 1560 | 252 | 9 | 75 % | 60 - 90% |
| F - Sphagnum magellanicum | MUE | 2888 | 154 | 17 | 75 % | 25 - 75% |

| **S2 C** | A | B | C | D | E | F |
| --- | --- | --- | --- | --- | --- | --- |
| A |  |  |  |  |  |  |
| B | 195 |  |  |  |  |  |
| C | 11 | 20 |  |  |  |  |
| D | 10 | 19 | 84 |  |  |  |
| E | 12 | 25 | 89 | 100 |  |  |
| F | 12 | 20 | 81 | 94 | 113 |  |
